# Quasi-species prevalence and clinical impact of evolving SARS-CoV-2 lineages in European COVID-19 cohorts, January 2020 to February 2022

**DOI:** 10.2807/1560-7917.ES.2025.30.10.2400038

**Published:** 2025-03-13

**Authors:** Matilda Berkell, Anna Górska, Mathias Smet, Delphine Bachelet, Elisa Gentilotti, Mariana Guedes, Anna Maria Franco-Yusti, Fulvia Mazzaferri, Erley Lizarazo Forero, Veerle Matheeussen, Benoit Visseaux, Zaira R. Palacios-Baena, Natascia Caroccia, Aline-Marie Florence, Charlotte Charpentier, Coretta van Leer, Maddalena Giannella, Alex W. Friedrich, Jesús Rodríguez-Baño, Jade Ghosn, ORCHESTRA working group, Samir Kumar-Singh, Cedric Laouénan, Evelina Tacconelli, Surbhi Malhotra-Kumar, ORCHESTRA working group, Elda Righi, Alessia Savoldi, Nadhem Lafhej, Basil Britto Xavier, Christine Lammens, Davide Gibellini, Michela Conti, Carmine Cutone, Filippo Cioli Puviani, Monica Compri, Romain Coppée, Reyene Menidjel, Maria Giulia Caponcello, Paula Olivare Navarro, Patricia Pérez-Palacios, Maria Immaculada López-Hernández

**Affiliations:** 1 Laboratory of Medical Microbiology, Vaccine & Infectious Disease Institute, University of Antwerp, Antwerp, Belgium; 2 Molecular Pathology Group, Cell Biology & Histology, Faculty of Medicine and Health Sciences, University of Antwerp, Antwerp, Belgium; 3 Division of Infectious Diseases, Department of Diagnostics and Public Health, University of Verona, Verona, Italy; 4 Université Paris Cité and Université Sorbonne Paris Nord, Inserm, IAME, Paris, France; 5 AP-HP Nord, Hôpital Bichat, Department of Epidemiology Biostatistics and Clinical Research, Paris, France; 6 Service de Virologie, AP-HP, Hôpital Bichat–Claude Bernard, Paris, France; 7 University of Groningen, University Medical Center Groningen, Department of Medical Microbiology and Infection Prevention, Groningen, The Netherlands; 8 Infectious Diseases and Microbiology Unit, University Hospital Virgen Macarena, Department of Medicine, University of Seville, Biomedicine Institute of Seville/CSIC, Seville, Spain; 9 CIBERINFEC, Instituto de Salud Carlos III, Madrid, Spain; 10 Infectious Diseases Unit, Department for Integrated Infectious Risk Management, IRCCS Azienda Ospedaliero-Universitaria di Bologna, Bologna, Italy; 11 Department of Medical and Surgical Sciences, Alma Mater Studiorum, University of Bologna, Bologna, Italy; 12 AP-HP Nord, Hôpital Bichat, Department of Infectious and Tropical Diseases, Paris, France; * Shared first author; ** Shared second author; *** Shared senior author

**Keywords:** Genome evolution, SNV, insertions, deletions, coronavirus, quasi-species, Italy, RNA secondary structure, mutations, mutation rate, ORCHESTRA, The Netherlands, Spain, France

## Abstract

**Background:**

Evolution of SARS-CoV-2 is continuous.

**Aim:**

Between 01/2020 and 02/2022, we studied SARS-CoV-2 variant epidemiology, evolution and association with COVID-19 severity.

**Methods:**

In nasopharyngeal swabs of COVID-19 patients (n = 1,762) from France, Italy, Spain, and the Netherlands, SARS-CoV-2 was investigated by reverse transcription-quantitative PCR and whole-genome sequencing, and the virus variant/lineage (NextStrain/Pangolin) was determined. Patients’ demographic and clinical details were recorded. Associations between mild/moderate or severe COVID-19 and SARS-CoV-2 variants and patient characteristics were assessed by logistic regression. Rates and genomic locations of mutations, as well as quasi-species distribution (≥ 2 heterogeneous positions, ≥ 50× coverage) were estimated based on 1,332 high-quality sequences.

**Results:**

Overall, 11 SARS-CoV-2 clades infected 1,762 study patients of median age 59 years (interquartile range (IQR): 45–73), with 52.5% (n = 925) being male. In total, 101 non-synonymous substitutions/insertions correlated with disease prognosis (severe, n = 27; mild-to-moderate, n = 74). Several hotspots (mutation rates ≥ 85%) occurred in Alpha, Delta, and Omicron variants of concern (VOCs) but none in pre-Alpha strains. Four hotspots were retained across all study variants, including spike:D614G. Average number of mutations per open-reading-frame (ORF) increased in the spike gene (average < 5 per genome in January 2020 to > 15 in 2022), but remained stable in ORF1ab, membrane, and nucleocapsid genes. Quasi-species were most prevalent in 20A/EU2 (48.9%), 20E/EU1 (48.6%), 20A (38.8%), and 21K/Omicron (36.1%) infections. Immunocompromised status and age (≥ 60 years), while associated with severe COVID-19 or death irrespective of variant (odds ratio (OR): 1.60–2.25; p ≤ 0.014), did not affect quasi-species’ prevalence (p > 0.05).

**Conclusion:**

Specific mutations correlate with COVID-19 severity. Quasi-species potentially shaping VOCs’ emergence are relevant to consider.

**Key public health message**

**What did you want to address in this study and why?**

In COVID-19 patients from different European countries, we studied SARS-CoV-2 evolution and how SARS-CoV-2 variants, both at clade level and through specific genetic mutations, impact disease presentation (mild or severe). Quasi-species (i.e. slightly differing forms of a recognised variant co-infecting a patient) prevalence was also investigated. The overarching aim was to identify clinical and viral genetic markers associated with COVID-19 severity.

**What have we learnt from this study?**

Mutation rates increased steeply with emergence of variants of concern (VOCs) and were remarkably variable across sub-genomic regions, compared with pre-Alpha variants. Only four hotspots (i.e. positions in the genome where average mutation rates exceed 85.0%) were found common to all variants. Being immunocompromised was associated with severe disease, as were 27 mutations. Quasi-species in COVID-19 patients were commonly found.

**What are the implications of your findings for public health?**

The frequent occurrence of quasi-species might influence associations between variants and disease severity as well as therapeutic and vaccination outcomes. We thus suggest analysing these additionally to the dominant infecting variant, e.g. by representing mutation probabilities or prevalence within a patient sample. This would enable inclusion of minority variants and mutations in future molecular epidemiological and viral transmission studies.

## Introduction

Since its emergence in late 2019, severe acute respiratory syndrome coronavirus 2 (SARS-CoV‑2) has continuously evolved. Mutations in the virus have resulted in increased transmission and infectivity as well as evasion of neutralising antibodies during SARS-CoV-2 infection and COVID-19. Despite its proofreading replication machinery, SARS-CoV-2 presented globally with two to four new mutations monthly [1]. These led to the emergence of variants of concern (VOCs) such as Alpha, Delta, and Omicron [2-5]. The variants coincided with diverse clinical presentation of COVID-19, including severe disease as well as more admissions to intensive care units (ICUs) (Delta) [6], and in some cases, increased mortality rates (Alpha) [7,8]. Some VOCs had increased transmission rates (Delta, Omicron) with infected patients having higher viral loads [9,10], while others coincided with higher rates of breakthrough infection (Delta, Omicron) [11,12], or improved disease outcome in terms of decreased hospitalisation and death rates (Omicron) [13,14].

During infection, SARS-CoV-2 is also known to form ‘quasi-species’. Quasi-species are viral sub-populations with one or several single nucleotide variations (SNVs) occurring at a given genome position. This phenomenon likely arises due to the inherent mutation rate of the virus, as well as in response to external stimuli like vaccination or therapy, as evidenced by emergence of resistant mutants to an anti-SARS-CoV-2 monoclonal antibody or antiviral therapy [15-18]. Moreover, previous studies have shown that immunocompromised individuals and patients with chronic COVID-19 suffer prolonged illness with higher viral loads, experience longer duration of viral shedding, and serve as a reservoir where the virus can mutate in vivo and be transmitted further [16,17,19-21]. While the epidemiological success of SARS-CoV-2 VOCs has been shown to be due to their enhanced transmissibility (Alpha, Delta, and Omicron) [8,9,22,23], or due to their high infectivity and immune evasion (Omicron) [3], viral genetic factors underlying infection severity and corresponding case-fatality rates remain incompletely understood. Mutations outside the SARS-CoV-2 spike (S) gene have been suggested to enhance virulence [24-26], but overall, little is known about the exact contribution of point mutations to disease severity to date.

To understand temporal emergence and evolution of SARS-CoV-2 variants and how this impacts COVID-19 severity and patient outcome, clinical data collection and viral variant sequencing were conducted and analysed for 1,762 patients diagnosed with COVID-19 in four European countries during January 2020–February 2022 that participated in the ongoing H2020 ORCHESTRA project. Along with studying the evolvability of the virus in a dynamic fitness landscape, we also aimed to study the prevalence of quasi-species and identify clinical and viral genetic markers associated with COVID-19 severity.

## Methods

### Study design

**Figure 1:**
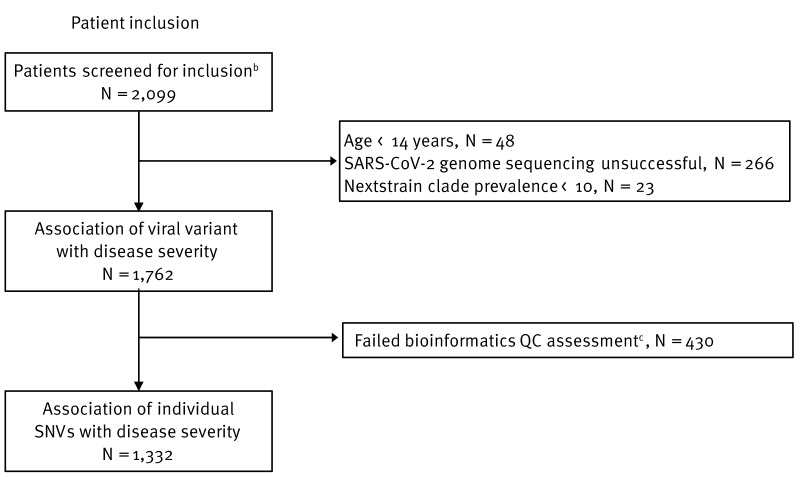
Flowchart illustrating steps to include patients and samples in the study in four European countries^a^, January 2020–February 2022 (n = 2,099 patients screened)

QC: quality control; SARS-CoV-2: severe acute respiratory coronavirus 2; SNV: single nucleotide variation.

^a^ The countries included Italy (Bologna and Verona), the Netherlands (Groningen), Spain (Andalucía), and France (Paris region).

^b^ Mildly, moderately, and severely ill COVID-19 patients diagnosed during January 2020—February 2022 (n = 2,099) were screened for inclusion.

^c^ Bioinformatics QC assessments included: assembly QC, mutation detection, and ≥ 60% coverage of detected mutations in the sample.

In this observational study, in- and out-patients presenting with COVID-19 symptoms were sampled (nasopharyngeal swab), either at the time of hospital admission or consultation with the general practitioner. The patients were enrolled between 29 January 2020 and 28 February 2022 in a multicentre study within the H2020-funded ORCHESTRA project (Connecting European Cohorts to Increase Common and Effective Response to SARS-CoV-2 Pandemic, https://orchestra-cohort.eu/). Samples considered for this study were collected as part of routine clinical practice in Italy (Bologna and Verona), the Netherlands (Groningen), Spain (Andalucía), and France (Paris region) within work package (WP)2, which comprised enrolling and sampling symptomatic COVID-19 patients that were analysed by WP6 dedicated to laboratory analyses of SARS-CoV-2 in ORCHESTRA. A total of 2,099 COVID-19 patients testing positive for SARS-CoV-2 by reverse transcription-quantitative (RT-q)PCR and with a stored nasopharyngeal swab sample were screened for inclusion in this study (Figure 1).

Inclusion criteria for all cohorts were: (i) testing positive for SARS-CoV-2 in RT-qPCR; (ii) availability of a stored nasopharyngeal swab sample from the acute phase of SARS-CoV-2 infection; (iii) availability of information on disease severity classified as mild, moderate, or severe COVID-19 as defined by the World Health Organization (WHO) clinical progression scale [27]; and (iv) availability of survival data within 1 month after diagnosis. The cohort-specific inclusion criteria are described in the Inclusion Criteria and Ethical Approval section in the Supplementary Information. Patients were excluded based on: (i) patient age < 14 years; (ii) unsuccessful SARS-CoV-2 genome sequencing; and (iii) overall SARS-CoV-2 NextStrain clade prevalence < 10 in the overall study population. Ultimately, a final study population of 1,762 patients was analysed further (Figure 1).

### Patient characteristics recorded and COVID-19 vaccination campaigns

For all patients in this study, we included (i) socio-demographical characteristics (i.e. age, biological sex (male/female), country of origin, information on the degree of COVID-19 severity (mild, moderate or severe illness), and vaccination status); (ii) comorbidities (i.e. diabetes, history of illness such as auto-inflammatory disease, cancer, cardiovascular disease, chronic kidney disease including dialysis, chronic liver disease, chronic respiratory disease, human immunodeficiency virus (HIV), immunosuppressive therapy and transplant; (iii) neurological and psychiatric disorders; (iv) COVID-19 treatments (i.e. oxygen therapy, anticoagulants, steroids, antibiotics); (v) complications (acute renal injury and cardiac, embolic, gastrointestinal, neurological or pulmonary events); and (vi) infecting SARS-CoV-2 variant for statistical analyses.

COVID-19 vaccination campaigns started on 27 December 2021 in the Netherlands, and on 10 January 2021 in Italy, France, and Spain. Vaccine doses received ≤ 14 days before COVID-19 diagnosis were not considered.

### Sample collection, analysis, and variant determination

Nasopharyngeal swab samples were collected in universal transport medium as part of routine COVID-19 diagnostic procedures at each local site and stored at −80 °C before shipment to one of the three analysing laboratories in this study: (i) Laboratory of Medical Microbiology, University of Antwerp, Antwerp, Belgium; (ii) Laboratory of Virology of the Bichat Claude Bernard hospital, Paris, France; and (iii) Department of Medical Microbiology and Infection Control, University Medical Center Groningen, Groningen, the Netherlands.

Nasopharyngeal swabs were subjected to RNA extraction, SARS-CoV-2 RT-qPCR, and viral whole genome sequencing (Oxford Nanopore Technologies or Illumina NextSeq/MiSeq). Classification of SARS-CoV-2 variants and lineages was performed using Nextclade v.2.2.0 (https://clades.nextstrain.org) and Pangolin v1.9 (4.0.6) (https://pangolin.cog-uk.io), respectively. Details are described in the Supplemental Methods section of the Supplementary Information.

### Bioinformatic analysis

To study dynamics of the SARS-CoV-2 genome across different variants over time, and to associate disease severity with synonymous and non-synonymous SNVs identified in this study, mutations as compared with the Wuhan-Hu-1 genome sequence were extracted through two bioinformatic pipelines: (i) a nucleotide (nt) based pipeline and (ii) a protein-based pipeline as described in Supplementary Figure 1. The Wuhan-Hu-1 genome (GenBank: NC_045512.2) was used as reference for all analyses. Coding regions of structural and non-structural proteins (nsps) used in this study were based on the Wuhan-Hu-1 sequence as described by Finkel et al. [28] and are enumerated in Supplementary Table 1.

#### Protein-based pipeline for single nucleotide variation identification

Long reads (Oxford Nanopore Technologies) were mapped against the human reference genome (GenBank: GRCh38) using minimap2 [29] v.2.17. Unmapped reads were extracted using SAMtools [30] v.1.9 and subsequently assembled using Rebaler v0.2.0 (https://github.com/rrwick/Rebaler) in a reference-guided manner. Short reads were trimmed and filtered based on quality by seqtk v1.2-r94 (https://github.com/lh3/seqtk) as indicated by FastQC [31]. Next, trimmed short reads were mapped against the human reference genome with Bowtie2 [32] v. 2.3.4.1. Unmapped reads were assembled with SPAdes [33] v.3.11.1 and aligned to the Wuhan-Hu-1 reference genome with RagTag [34]. Final assemblies used for downstream analyses were obtained by mapping raw or trimmed reads to the SARS-CoV-2 assembly from the same patient using minimap2 (long reads) or Bowtie2 (short reads) followed by polishing with Pilon [35] v.1.23.

Genes were predicted with GeneMarkS [36] v.4.32 for assembled and polished genomes, and translated proteins were extracted and aligned against the National Center for Biotechnology Information (NCBI) non-redundant (nr) database using Diamond [37] v.2.0.14. Alignments defined by a query coverage ≥ 98% (query and target), the smallest e-value, and highest identity were considered. Due to the overlap between open reading frame (ORF)1a and ORF1b, Diamond alignment was performed individually against reference sequences of all individual parts of ORF1ab. Finally, multiple-sequence alignments for each individual protein were computed with MAFFT [38] v7.490. Alignment processing was performed using Python v3.9, and visualisations were created using Matplotlib v.3.3.3 (https://ieeexplore.ieee.org/document/4160265), followed by image annotation in Inkscape 1.1 (23 May 2021). For downstream statistical analyses, only substitutions, insertions, and deletions present in ≥ 10% of sequences were considered, and neighbouring SNVs were merged.

#### Nucleotide-based pipeline for single nucleotide variation identification

Reads were mapped against the Wuhan-Hu-1 genome using Bowtie2 or minimap2 as described above, followed by computation of pileups and parsing using samtools v.1.9 and pileup2base (https://github.com/riverlee/pileup2base), respectively. Per-base mutation rates were computed for positions with a coverage ≥ 10 as:

Per-base mutation rate = 1 – (proportion of reads carrying the same nt as Wuhan-Hu-1).

Sequences were grouped by infecting SARS-CoV-2 variant, and the average per-base mutation rate was computed for clades with > 5 sequences each. Positions with an average mutation rate ≥ 15% in at least one variant, as well as positions with mutation rates between 5% and 15% present in at least two variants were considered for further analysis. Finally, observed SNVs were compared with those identified by the protein-based SNV identification pipeline, and to mutations described previously in literature (https://outbreak.info/ [39,40]).

#### Identification of within-patient SARS-CoV-2 populations (quasi-species)

Read mapping against the Wuhan-Hu-1 reference sequence enabled identification of SNVs in individual genome positions within a single sample. At each position of the SARS-CoV-2 genome with ≥ 50× coverage, pileups computed as previously described were represented as relative proportions of bases A, T, C, or G, rounding proportions to one decimal, and grouping positions with identical patterns based on the reasoning that if enough positions shared the same proportion of mutations, this would suggest existence of a second minority subvariant (quasi-species) within the sample that was lost during assembly. Positions with patterns 70/30%, 60/40%, and 50/50% (i.e. heterogeneous positions) were of interest where one of the nts correspond to its assembled sequence and one constitutes a mutation. The latter were cross-referenced with the mutations database (https://outbreak.info/, downloaded on 09 June 2023) considering mutations present in 1% of sequences in this database to ensure the largest inclusivity. Next, each potential mutation was mapped against Pangolin lineages acquired from the database. Subsequently, the list of lineages was filtered by removing descending lineages if the parent was present. This way, for each sample and pattern, a list of mutations was translated into a list of potential lineages. Finally, the variant with highest probability estimated by hypergeometric distribution was selected [41]. Presence of quasi-species was noted if > 10 heterogeneous positions per variant were observed. 

#### Secondary structure analysis

Secondary structure of the SARS-CoV-2 genome, reactivity, and level of determined structure was analysed as described by Tavares et al. [42].

### Statistical analysis

Data summary, statistical analyses, and visualisation of patient characteristics was performed using R [43] v4.1.2. Patient characteristics were described in the whole population, by country, and by infecting SARS-CoV-2 variant. Continuous variables were described as medians and interquartile ranges (IQRs). Categorical variables were described by numbers and percentages of patients with available data. Quantification cycle (Cq) values from SARS-CoV-2 RT-qPCRs with different gene targets performed in the study were analysed in priority of: envelope (E) protein, ORF1ab, nucleocapsid (N) protein, S protein, and non-specified gene target. Comparisons of viral loads defined by Cq values were performed between patients infected by different SARS-CoV-2 variants using pairwise Wilcoxon rank-sum tests followed by Bonferroni post-hoc correction.

The associations between each patient’s characteristics (age, sex, comorbidities, vaccination status), infecting SARS-CoV-2 variants, and the degree of disease severity were analysed using a mixed bivariate logistic regression model including a random intercept for country of origin.

For association of SARS-CoV-2 variants and individual SNVs in the SARS-CoV-2 genome with COVID-19 severity, the WHO clinical progression scale [27] was used to classify patients as (i) mildly ill (ambulatory patients); (ii) moderately ill (hospitalised with no oxygen or oxygen by mask or nasal prongs); (iii) severely ill (hospitalised patients receiving oxygen by non-invasive ventilation (NIV) or high flow and/or intubation, and mechanical ventilation or/and vasopressors, dialysis, extracorporeal membrane oxygenation (ECMO)); or dead. As no Omicron-infected patients died in the study, disease severity for all COVID-19 patients included here was divided into two categories: (i) severe disease or death, and (ii) mild or moderate COVID-19.

For multivariate analysis, effect of viral variants (Nextstrain classification) on disease severity was adjusted based on age, sex, number of comorbidities (< 2 vs ≥ 2), and number of vaccine doses received.

To assess if there is a risk of developing quasi-species with older age (≥ 60 years) or immunocompromised status, odds ratios were calculated using the crosstabs procedure in SPSS (v29.0.1.0), with p values derived from the chi-squared test of independence. Immunocompromised status was defined as having a medically recognised immunocompromising condition or receiving immunosuppressants for cancer, solid organ transplant, or similar conditions. Patients receiving corticosteroids (e.g. 6 mg dexamethasone) were not classified as immunocompromised in this analysis. 

Association of synonymous and non-synonymous SNVs with disease severity were individually assessed using bivariate mixed logistic regression models including a random intercept for country of origin. The p values were corrected per gene using false discovery rate (FDR) post-hoc correction.

All performed tests were two-sided and a (corrected) p value < 0.05 was considered significant.

## Results

### Characteristics of COVID-19 patients

As part of the multi-centre study, 2,099 symptomatic patients diagnosed with COVID-19 during January 2020–February 2022 were screened for inclusion (Figure 1). Overall, 1,762 patients with a successfully sequenced SARS-CoV-2 genome were selected for further analysis, where 61.9%, 17.0%, and 7.7%, respectively, presented with mild (n = 1,090), moderate (n = 299), and severe (n = 136) COVID-19 as defined by the WHO clinical disease progression scale [27]; 13.5% died during the study period (n = 237) (Table). Overall, 38.4% patients were hospitalised (676/1,762; 237 deaths, 136 severely, 299 moderately and four mildly ill), of whom 30.4% (201/662, data missing for 14 patients) were admitted to the ICU. Most enrolled patients in the study originated from Italy (n = 1,159), followed by the Netherlands (n = 353), Spain (n = 149), and France (n = 101). Patients had a median age of 59 years (IQR: 45–73), 52.5% (n = 925) were male, and the most common comorbidities were cardiovascular disease (44.0%), diabetes (16.0%), and chronic pulmonary disease (13.9%). Patient characteristics are described in the Table.

**Table:** Characteristics of COVID-19 patients enrolled in the study in four European countries^a^, January 2020–February 2022 (n = 1,762 patients)

Patient characteristics		Missing values		Overall (n = 1,762)			Italy (n = 1,159)			The Netherlands (n = 353)			Spain (n = 149)			France (n = 101)			p value
		N	%^b^	N	Total	%	N	Total	%	N	Total	%	N	Total	%	N	Total	%	
**Demographic characteristics and viral load**																			
Male sex, n (%)		0	0	925	1,762	52	609	1,159	53	174	353	49	72	149	48	70	101	69	0.003
Age, median (IQR)		0	0	59 (45–73)			63 (48–75)			49 (33–64)			53 (38–69)			65 (56–72)			< 0.001
Viral loads (Cq), median (IQR)		231	13	18.8 (15.3–22.3)			18.0 (15.0–21.8)			23.0 (18.0–27.0)			19.4 (16.7–21.3)			22.2 (18.4–24.8)			< 0.001
**Comorbidities**																			
Cardiovascular disease, n (%)		531	30	542	1,231	44	459	1,041	44	na	na	na	57	149	38	26	41	63	0.021
Diabetes, n (%)	No	434	25	1,115	1,328	84	963	1,141	84	na	na	na	128	149	86	24	38	63	0.011
	Type 1			9	1,328	1	9	1,141	1	na	na	na	0	149	0	0	38	0	
	Type 2			200	1,328	15	165	1,141	14	na	na	na	21	149	14	14	38	37	
	Other type			4	1,328	0	4	1,141	0	na	na	na	0	149	0	0	38	0	
Chronic pulmonary disease, n (%)		446	25	183	1,316	14	155	1,126	14	na	na	na	17	149	11	11	41	27	0.039
CKD, n (%)	On dialysis	448	25	26	1,314	2	23	1,133	2	na	na	na	2	149	1	1	32	3	0.019
	Without dialysis			76	1,314	6	70	1,133	6	na	na	na	5	149	3	1	32	3	
Chronic liver disease, n (%)		561	32	33	1,201	3	24	961	2	na	na	na	4	149	3	5	91	5	0.247
Psychiatric disorder, n (%)		423	24	90	1,339	7	70	1,149	6	na	na	na	20	149	13	na	na	na	< 0.001
Neurological disorder, n (%)		427	24	127	1,335	10	119	1,145	10	na	na	na	8	149	5	na	na	na	0.016
Autoinflammatory disease, n (%)		377	21	72	1,385	5	55	1,159	5	na	na	na	14	149	9	3	78	4	0.045
Immunosuppression, n (%)		474	27	159	1,288	12	148	1,098	13	na	na	na	0	149	0	11	45	27	< 0.001
HIV, n (%)		969	55	6	793	1	4	551	1	na	na	na	0	149	0	2	93	2	0.07
Transplant, n (%)		705	40	36	1,057	3	8	536	1	25	286	9	0	149	0	3	86	3	< 0.001
Cancer, n (%)		964	55	80	798	10	52	500	10	11	54	20	7	149	5	10	95	11	0.011
**Viral variants (NextStrain)**																			
19A, n (%)		0	0	12	1,762	1	0	1,159	0	9	353	3	0	149	0	3	101	3	< 0.001
20A, n (%)		0	0	238	1,762	14	136	1,159	12	46	353	13	31	149	21	25	101	25	
20A/EU2, n (%)		0	0	56	1,762	3	28	1,159	2	15	353	4	1	149	1	12	101	12	
20B, n (%)		0	0	71	1,762	4	53	1,159	5	8	353	2	4	149	3	6	101	6	
20C, n (%)		0	0	23	1,762	1	1	1,159	0	9	353	3	3	149	2	10	101	10	
20E/EU1, n (%)		0	0	279	1,762	16	172	1,159	15	29	353	8	74	149	50	4	101	4	
20I/Alpha, n (%)		0	0	290	1,762	16	203	1,159	18	36	353	10	36	149	24	15	101	15	
21I/Delta, n (%)		0	0	60	1,762	3	51	1,159	4	8	353	2	0	149	0	1	101	1	
21J/Delta, n (%)		0	0	507	1,762	29	381	1,159	33	114	353	32	0	149	0	12	101	12	
21K/Omicron, n (%)		0	0	215	1,762	12	127	1,159	11	78	353	22	0	149	0	10	101	10	
21L/Omicron, n (%)		0	0	11	1,762	1	7	1,159	1	1	353	0	0	149	0	3	101	3	
**COVID-19 severity**																			
WHO-scale disease severity, n (%)^c^	Mild	0	0	1,090	1,762	62	767	1,159	66	224	353	63	99	149	66	0	101	0	< 0.001
	Moderate	0	0	299	1,762	17	184	1,159	16	39	353	11	27	149	18	49	101	49	
	Severe	0	0	136	1,762	8	60	1,159	5	37	353	10	6	149	4	33	101	33	
	Death	0	0	237	1,762	13	148	1,159	13	53	353	15	17	149	11	19	101	19	
Hospitalisation, n (%)		0	0	676	1,762	38	393	1,159	34	132	353	37	50	149	34	101	101	100	< 0.001
Duration in days, median (IQR)		253	37^d^	10 (6–21)			11 (7–21)			8 (4–17)			9 (6–20)			11 (6–26)			0.044
ICU admission, n (%)		14	2^d^	201	662	30	74	387	19	80	131	61	6	49	12	41	95	43	< 0.001
Duration in days, median (IQR)		42	21^d^	11 (7–21)			10 (7–21)			13 (7–20)			23 (9–44)			12 (6–15)			0.583
**Treatment**																			
Corticosteroids, n (%)		494	28	357	1,268	28	299	1,063	28	na	na	na	32	148	22	26	57	46	0.003
Antiviral agent, n (%)		420	24	166	1,342	12	127	1,105	11	na	na	na	19	148	13	20	89	22	0.01
Immunomodulator, n (%)		472	27	56	1,290	4	50	1,101	5	na	na	na	6	147	4	0	42	0	0.361
Hydroxychloroquine, n (%)		365	21	82	1,397	6	54	1,159	5	na	na	na	19	148	13	9	90	10	< 0.001
Azithromycin, n (%)		414	23	64	1,348	5	60	1,159	5	na	na	na	4	148	3	0	41	0	0.143
Monoclonal antibodies, n (%)	None	644	37	660	1,118	59	512	970	53	na	na	na	148	148	100	na	na	na	< 0.001
	Bamlanivimab/ etesevimab	644	37	224	1,118	20	224	970	23	na	na	na	0	148	0	na	na	na	
	Casirivimab/ imdevimab	644	37	218	1,118	19	218	970	22	na	na	na	0	148	0	na	na	na	
	Other	644	37	16	1,118	1	16	970	2	na	na	na	0	148	0	na	na	na	
Antibiotics, n (%)		490	28	327	1,272	26	274	1,071	26	na	na	na	17	148	11	36	53	68	< 0.001
Oxygen, n (%)		445	25	417	1,317	32	317	1,079	29	na	na	na	36	149	24	64	89	72	< 0.001
**Anti-SARS-CoV-2 vaccination (> 2 weeks after dose)**																			
Unvaccinated, n (%)		408	23	985	1,354	73	626	953	66	165	184	90	134	148	91	60	69	87	< 0.001
1 dose, n (%)		408	23	42	1,354	3	29	953	3	1	184	1	10	148	7	2	69	3	
≥ 2 doses or 1 dose of Ad26.COV2.S, n (%)		408	23	327	1,354	24	298	953	31	18	184	10	4	148	3	7	69	10	
**Complications**																			
Pulmonary events, n (%)		496	28	16	1,266	1	14	1,081	1	na	na	na	1	149	1	1	36	3	0.58
Cardiac events, n (%)		498	28	62	1,264	5	58	1,079	5	na	na	na	1	149	1	3	36	8	0.028
Embolic events, n (%)		498	28	9	1,264	1	5	1,079	0	na	na	na	2	149	1	2	36	6	0.001
Neurological events, n (%)		491	28	8	1,271	1	4	1,084	0	na	na	na	2	149	1	0	36	0	0.245
Acute renal injury, n (%)		413	23	21	1,349	2	13	1,159	1	na	na	na	4	149	3	4	41	10	< 0.001
Gastrointestinal events, n (%)		493	28	12	1,269	1	11	1,084	1	na	na	na	0	149	0	1	36	3	0.251

Ad26.COV2.S: Jcovden (Ad26.COV2-S, Janssen-Cilag International NV) vaccine; CKD: chronic kidney disease; Cq: quantification cycle; ICU: intensive care unit; IQR: inter-quartile range; na: data not recorded; SARS-CoV-2: severe acute respiratory syndrome coronavirus 2; WHO: World Health Organization.

^a^ The countries included Italy (Bologna and Verona), the Netherlands (Groningen), Spain (Andalucía), and France (Paris region).

^b^ The denominator for the percentage is 1,762 unless otherwise specified (see footnote d).

^c^ Disease severity was classified according to the WHO disease severity scale [27].

^d^ The percentages of missing data are based on the number of people hospitalised (i.e. the denominator is 676).

### Epidemiology of SARS-CoV-2 variants and COVID-19

**Figure 2:**
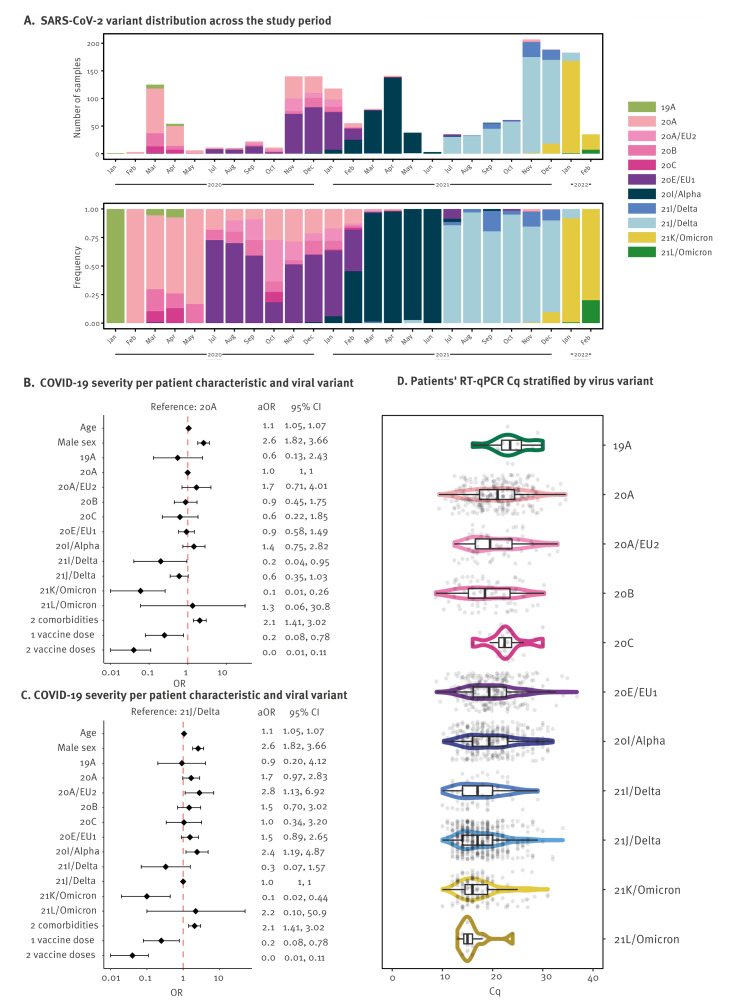
(A) Distribution of infecting SARS-CoV-2 variants, (B, C) COVID-19 severity by patients’ demographic characteristics and variant, and (D) viral loads by variant in patients from four European countries^a^, January 2020–February 2022 (n = 1,762 patients)

Eleven different SARS-CoV-2 variants were observed among the 1,762 patients in this study, of which 21J/Delta (28.8%, n = 507), 20I/Alpha (16.5%, n = 290), 20E/EU1 (15.8%, n = 279), 20A (13.5%, n = 238), and 21K/Omicron (12.2%, n = 215) were most frequent (Table, Figure 2A); this is also illustrated in Supplementary Figure 2. During March–April 2020, higher diversity of SARS-CoV-2 variants was observed in patients from the Netherlands and France (predominantly 19A, 20A, 20B, 20C), while patients from Italy and Spain primarily experienced 20A infections (Supplementary Figure 2). Several Pangolin lineages were associated with variant 20A (primarily B.1, B.1.221, B.1.416.1). With the emergence of 20E/EU1 in Spain in June 2020 [44], which was later observed in all participating countries (July 2020–January 2021), the number of circulating lineages decreased as this variant was primarily associated with the B.1.177 lineage (Figure 2A), as shown in Supplementary Table 2. Similarly, a single or few lineage(s) dominated with the emergence of VOCs, including 20I/Alpha (B.1.1.7; February–June 2021) and 21I/Delta (AY.9.2). This trend was reversed with the emergence of 21J/Delta (namely AY.43, AY.4, AY.122; July–December 2021) and 21K/Omicron (BA.1.1, BA.1.17.2, BA.1) that again showed higher prevalence of individual lineages than predecessor VOCs.

aOR: adjusted odds ratios; Cq: quantification cycle; OR: odds ratio; RT-qPCR: reverse transcription quantitative PCR; SARS-CoV-2: severe acute respiratory syndrome coronavirus 2.

^a^ The countries included Italy (Bologna and Verona), the Netherlands (Groningen), Spain (Andalucía), and France (Paris region).

(A) Distribution of infecting SARS-CoV-2 variants across the study period (n = 1,762). (B, C) Using (B) 20A or (C) 21J/Delta as reference, forest plots associate infecting SARS-CoV-2 variants and patient characteristics with COVID-19 severity respectively, displayed as adjusted ORs from mixed multivariable logistic regression model including a random intercept for country of origin and age, sex, infecting SARS-CoV-2 variants, number of comorbidities, received number of vaccine doses as fixed effects (n = 1,762). (D) Combined RT-qPCR Cq values for patients stratified by viral variant (n = 1,531). Viral loads inferred by combined Cq values were found to differ overall (Kruskal−Wallis, p < 2.2e-16). Supplementary Table 6 provides more detail on pairwise comparisons of viral loads between variants. Box plots show median (line), 25th–75th percentiles (box), and 5th–95th percentiles (whiskers). All data points are displayed. Violin plots implement a rotated kernel density plot on each side, adding information regarding distribution of the measured data; the width of the violin indicates the frequency.

Comparing characteristics of patients infected with different SARS-CoV-2 variants revealed a decreased disease severity over the study period (Figure 2B and C). Patients infected with 21K/Omicron (n = 215) were younger (56 years; 95% confidence interval (CI): 39−68 years) and less frequently male (41.9%; 90/215), as described in Supplementary Table 3. Mixed logistic regression modelling revealed an association between male sex and ≥ 2 comorbidities with higher odds of developing severe disease across all variants (odds ratio (OR): 2.60; 95% CI: 1.13−6.92, and OR: 2.1; 95% CI: 1.13−6.92, respectively). Infections due to 21K/Omicron were associated with a reduced risk of severe disease compared with 21J/Delta (adjusted OR (aOR): 0.10; 95% CI: 0.02−0.44) (Figure 2B and C) as reported previously [45], and also described in Supplementary Tables 4 and 5. In contrast, few pre-Delta variants, were associated with a higher risk of severe disease, such as 20A/EU2 (OR: 2.80; 95% CI: 1.13−6.92) and 20I/Alpha (OR: 2.41; 95% CI: 1.19−4.87) (Supplementary Table 3), as reported previously [46,47].

Viral loads differed between SARS-CoV-2 variants (Kruskal−Wallis, p < 2.2e-16); patients infected with Delta and Omicron variants had significantly higher viral loads compared with patients with pre-Delta variants (Figure 2D), as previously observed [17,48-50]; this is also shown in Supplementary Table 6. However, no differences were observed in Cq values between Delta- and Omicron-infected patients (p > 0.05), although some studies have shown that both BA.1 and BA.2 sub-variants result in higher viral loads than Delta [49]. Consistent with the findings that high viral loads of Delta and Omicron variants do not necessarily cause severe disease, we observed no appreciable risk of developing severe COVID-19 with high viral loads (OR: 1.03; 95% CI: 1.00−1.06, p = 0.045; Supplementary Table 4).

### SARS-CoV-2 VOCs display higher but variable mutation rates across sub-genomic regions compared with historical variants

**Figure 3:**
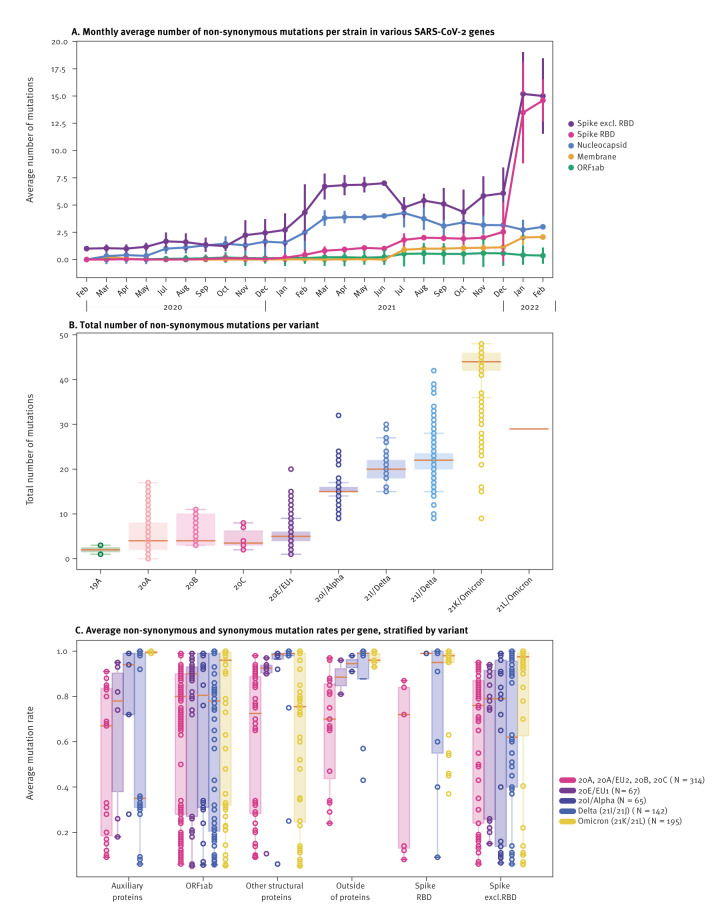
Evolution of the SARS-CoV-2 genome based on viral genetic sequences derived from patients from four European countries^a^, January 2020–February 2022 (n = 1,332)

To study the evolution of the SARS-CoV-2 genome over time, all SARS-CoV-2 sequenced here (n = 1,762) were mapped against the Wuhan-Hu-1 strain; of these, 1,332 sequences passed quality control as described in Figure 1, and were further analysed with the nt- and protein-based pipelines for in-depth SNV characterisation, as shown in Supplementary Figure 4. Overall, 325 mutated genome positions with 426 unique nt substitutions, insertions, or deletions were identified, as detailed in Supplementary Table 7. Maximum mutations mapped to the S gene (24.6%, 105/426), followed by nsp3 (12.4%, 53/426), N (12.0%, 51/426), and polymerase (5.6%, 24/426) genes across all lineages. While many S gene mutations were located within the receptor-binding domain (RBD) region, several were also located outside the S-RBD region, specifically in Omicron lineages (Figure 3). Of the identified mutations, 54.2% (231/426) were non-synonymous, impacting 208 positions in the SARS-CoV-2 genome.

Excl.: excluding; ORF: open reading frame; RBD: receptor binding domain; SARS-CoV-2: severe acute respiratory syndrome coronavirus 2.

^a^ The four countries (regions) in which the study was conducted included Italy (Bologna and Verona), the Netherlands (Groningen), Spain (Andalucía), and France (Paris region).

(A) Evolution of the average number of non-synonymous mutations observed per strain compared with the Wuhan reference sequence (NC_045512.2) for ORF1ab, membrane, nucleocapsid, spike RBD, and spike excluding RBD genes during the study period. Coloured dots represent average mutation numbers with error bars showing standard deviation. (B) Total number of non-synonymous mutations for each patient, stratified per variant; single sequences are represented by circles and their colour and position relative to the X axis correspond to the variant they belong to. Box plots show median (line), 25th–75th percentiles (box), and 5th–95th percentiles (whiskers). Note that only seven patients were infected by the 21K/Omicron variant. (C) Distribution of average nt mutation rates (synonymous and non-synonymous) per variant, stratified by genes. A single circle corresponds to the proportion of sequences carrying the variant. Outliers (median ± 1.5 times the interquartile range) were removed. The 19A sub-lineages is not shown due to sequence counts < 10.

When assessing average number of mutations per ORF, a drastic increase over time was observed in the S gene (from < 5 on average/genome in January 2020 to > 15 in 2022). In contrast, mutation numbers in ORF1ab, membrane, and N genes remained relatively stable throughout the study (Figure 3A).

Not surprisingly, this temporal increase in S gene mutations coincided with emergence of Alpha, Delta, and Omicron VOC lineages (Figure 3B). On the other hand, mutation rates across different ORFs varied widely among different variants (Figure 3C). For instance, ORF1ab, which encodes nsps important for viral replication, showed a wide distribution in mutation rates across all variants. In genes encoding structural proteins (S, membrane, N, E), mutations were widespread in Omicron and in historical variants (20A/B/C), with consistently high mutation rates across Delta (21I/J), 20I/Alpha, and 20E/EU1 variants (Figure 3C). Although Omicron (21K/L) variants exhibited the highest overall mutation rates (Figure 3B), these were mainly concentrated in auxiliary proteins (such as exonuclease, nsp, and helicase), the S gene (both in and outside of RBD), and in intergenic or non-coding genomic regions (Figure 3C).

### Mutation hotspots across the SARS-CoV-2 genome are lineage-specific

**Figure 4:**
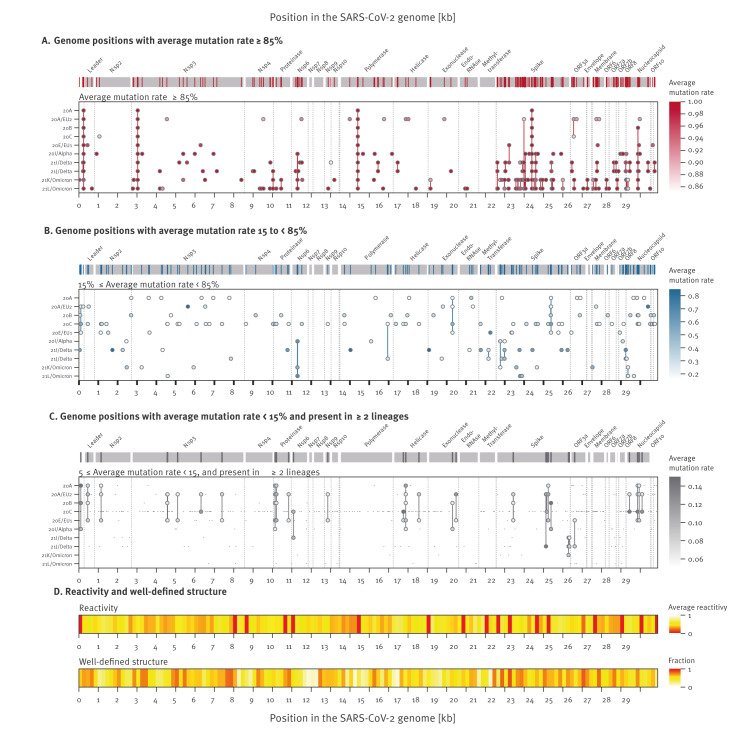
Mutation rates at different positions across the SARS-CoV-2 genome, January 2020–February 2022 (n = 1,332)

To analyse evolutionary patterns in the SARS-CoV-2 genome, mutation hotspots were identified as nt positions within a variant showing an average mutation rate of (i) ≥ 85%, (ii) 15 to < 85%, or (iii) 5 to < 15% (across at least two lineages) compared with Wuhan-Hu-1 (Figure 4; Supplementary Table 7). This is also illustrated in Supplementary Figures 4 and 5. The analysis of variant-specific mutation rate-dynamics over time (Figure 3A-C) revealed that the highest density of mutation hotspots (with an average ≥ 5.0%) was observed in the S (n = 80 mutation hotspots), N (n = 27), and nsp3 (n = 23) genes. Additionally, 14 mutation hotspots were identified in non-coding regions of the SARS-CoV-2 genome, with several of these hotspots being common across multiple variants.

SARS-CoV-2: severe acute respiratory coronavirus 2.

Nucleotide mutation hotspots were identified by read mapping against the Wuhan-Hu-1 SARS-CoV-2 reference genome (NC_045512.2). Each circle corresponds to a position on the SARS-CoV-2 genome where the average mutation rate for a variant was: (A) ≥ 85% among samples of a single variant, (B) 15 to < 85%, or (C) 5 to < 15% and present in at least two variants. The colour shade of each mutation hotspot (circle) determines its abundance and lines connect mutation hotspots found on the same positions across different variants. (D) Reactivity (SHAPE [62]) and proportion of well-defined secondary structure [63], i.e. high content of the paired nt and low entropy, across different genomic regions are presented as fractions (as first described by Tavares et al. [42]). We anticipated that sites with high reactivity and lower proportions of well-defined structure — indicated in yellow — would be more susceptible to mutations and would contain a greater number of mutation hotspots.

Four nt substitutions were identified with an average frequency exceeding 85.0% across all variants. These include: one non-coding substitution (C241T), two synonymous mutations in nsp3 (C3037T) and polymerase (C14408T) genes within ORF1ab, and one non-synonymous substitution (A23403G, S:D614G; Figure 4A, Supplementary Table 7). S:D614G was detected in the Alpha variant in April 2020, and has since been common for both Delta and Omicron lineages (https://outbreak.info). This mutation was initially detected together with S:del69–70, which in this study was consistently observed (≥ 95% frequency) in 20I/Alpha and Omicron (21K/L). Both S:D614G and S:del69–70 are known to increase transmissibility [10].

Mutation hotspots with an average mutation rate ≥ 85.0% across multiple lineages, specifically across Delta and Omicron lineages, were primarily observed within the S gene (Figure 4A). While some of the hotspots were found to be common for both Delta and Omicron variants, the majority were found to be either Delta or Omicron-specific [39,40]. Among Omicron variants (21K/21L), 48 mutated positions were shared, of which 35 were Omicron-specific. These included S:Q493R (A23040G), known to confer resistance to the monoclonal antibodies bamlanivimab and etesevimab [15,51,52], as well as S:S371P/F (T22673C/C22673T), S:S373P (T22679C), and S:S375F (C22686T), known to play a crucial role in stabilising S-RBD conformation in Omicron variants to counter the conformational alteration triggered by angiotensin-converting enzyme 2 (ACE2) binding [53]. Notably, nt positions with an average mutation rate ≥ 85.0% in Omicron lineages were observed at lower rates in earlier variants. Similarly, Delta variants (21I and 21J) shared 37 mutation hotspots, of which 28 were Delta-specific.

Variants of concern (Alpha, Delta, Omicron) were found to harbour many mutation hotspots with mutation rates ≥ 85.0% in this study, reflective of the increasing number of lineage-defining mutations in VOCs over time. In contrast, pre-Alpha variants either entirely lacked mutational hotspots or harboured hotspots with mutation rates within the range of 5 to < 85% (Figure 4B and C). Mapping of reads onto the reference genome enabled direct assessment of the proportion of reads carrying the mutation providing a continuous probability distribution rather than a simple probability of detection (i.e. present or absent). We show that the probability of having both mutations and mutational hotspots specific to variants increases when ordered by sample collection date. Supplementary Figure 4 shows the per-sample mutations ordered by time and coloured by variant. Mutation rates differed throughout the genome and were highest in the Receptor Binding Motif (RBM) in S-RBD.

To summarise, only four mutation hotspots were retained across all variants in the study, and most of the mutation hotspots specific to single lineages were observed in the Omicron- and Delta-associated lineages.

### Relationship between secondary structure and mutation rates across the SARS-CoV-2 genome

To compare mutation rates across SARS-CoV-2 genomic regions with differing RNA structural complexity, we mapped mutation hotspots to the RNA secondary structure, nt reactivity, and level of conservation [42]. Overall, 98 positions with an average mutation rate > 5.0% across all samples were observed in base-paired genomic regions, while 71 were observed in non-paired regions (Figure 4D). Mutation rates did not differ between the two regions, nor did they correlate with measurements of structural stability and nt reactivity as defined by Tavares et al. [42], as shown in Supplementary Figure 6A and B, indicating that nt accessibility or stability is not a factor influencing the mutation rate in the SARS-CoV-2 genome. Extended results from this section can be found in the Supplementary Information.

### Association of individual mutations with COVID-19 severity

Next, we attempted to associate individual mutations observed in this study with patient outcomes using bivariate logistic regression models. The nt-based pipeline discovered 221 non-synonymous mutations in coding sequences, of which 111 were already found by the protein-based-pipeline, and 10 mutations in non-coding sequences.

**Figure 5:**
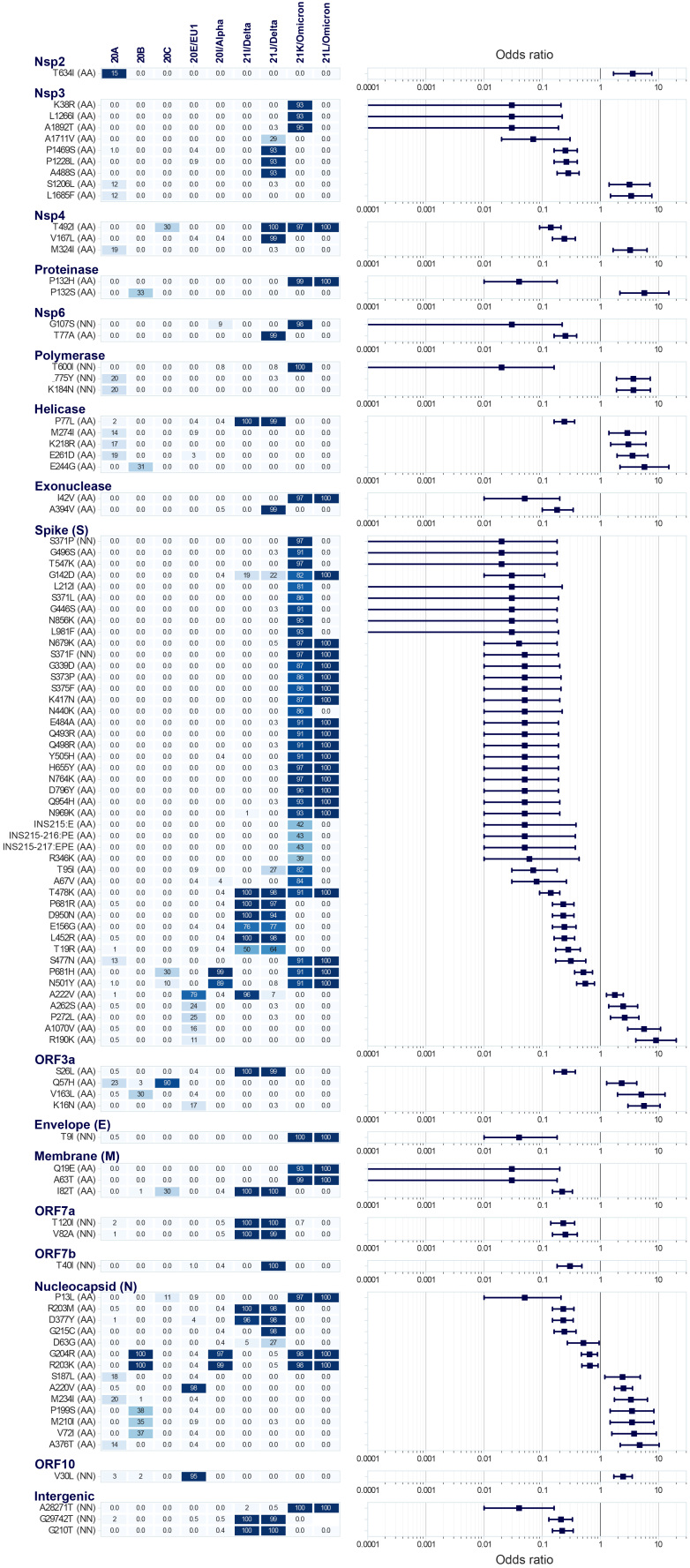
Association of non-synonymous mutations in the SARS-CoV-2 genome and COVID-19 severity, January 2020—February 2022 (n = 1,332 sequences)

AA: protein-based-pipeline-identified mutation; NN: nucleotide-based-pipeline-identified mutation; OR: odds ratio.

Using both protein- and nt-based pipelines in combination, across 1,332 patients, yielded 187 mutations, and 101 of the identified non-synonymous ones were found to be either positively (n = 27) or negatively (n = 74) associated with COVID-19 severity (FDR-corrected p value (*P_FDR_*) < 0.05, Figure 5), as also described in Supplementary Table 8 and 9. Of these, 61% (62/101) comprised mutations in structural proteins (S, N, membrane). Among the 101 mutations, 79 were identified with both SNV pipelines, whereas eight and 14 were identified only by the protein-based or the nt-based SNV pipeline, respectively. The protein-based pipeline is more robust, as it relied on de-novo assembly and subsequent multiple-sequence protein alignment, however, the nt-pipeline is more sensitive, and enables analysis of mutations in non-coding regions.

The left panel presents the percentage of genomes within each variant carrying mutations or insertions represented as a heatmap. Forest plots in the right panel show associations between individual mutations and disease in each protein or genomic region expressed as ORs and their 95% confidence interval. All identified mutations were included in assessing links with disease severity, but only mutations with a corrected p < 0.05 are displayed. Refer to Supplementary Table 8 for literature describing individual mutations. OR > 1.0 signifies association with severe COVID-19.

Among the total 87 non-synonymous mutations identified via the protein-based SNV identification pipeline, 24 showed a significant positive (OR > 1.0) and 63 negative (OR < 1.0) association with COVID-19 severity during acute/early infection, where 60 affected structural protein genes. Most of these (72/87) had previously been observed in at least 5% of identified SARS-CoV-2 strains (https://outbreak.info, accessed on 22 November 2022), while 15 were novel. 

Expectedly, most of the non-synonymous mutations (n = 42 substitutions and 3 insertions in protein-based pipeline, n = 2 in nt-based pipeline) significantly linked with disease severity were observed in the S gene. Of the 47, only five of the S protein substitutions were associated with severe COVID-19 (OR > 1.0, *P_FDR_* < 0.05). These were either affecting an N-terminal domain of the S2 subunit [54] (R190K (novel), A222V (20A/E), A262S, P272L (novel)) or located between the heptapeptide repeat sequence 1 and 2 (A1070V (novel)). S:A262S was observed mainly in 20E/EU1 strains in this study but is also reported in Omicron lineages (BA.5.10, https://outbreak.info/). Of the S gene mutations associated with mild disease, 16 were observed in S-RBD, 10 of which were in RBM and are present in Omicron lineages (see Results and Discussion in Supplementary Information). Of note, S:D614G, which was one of the four mutations present in all of the sequences studied here (Figure 4A), was also associated with mild disease severity.

Seven mutations in the N protein located either in the RNA-binding domain, the dimerisation domain, the C-terminus or affecting the linker-mediating interface with nsp3, were associated with up to three-fold higher odds of increased disease severity (Figure 5, Nucleocapsid panel). Only two substitutions in nsp3 and three in ORF3a were associated with severe COVID-19 (Figure 5, nsp3 and ORF3a panels). Extended results on identified mutations and links with disease severity are presented in the Results and Discussion of the Supplementary Information.

### Identification of quasi-species and mutational heterogeneity in COVID-19 patients

Next, we analysed prevalence of quasi-species expressed as minority variants during reference mapping. Each sample with characterised mutation hotspots (n = 1,332, > 50× coverage) was screened for genomic positions that had exactly two nts mapped with the same relative proportion of mutated nts (30–70%). Overall, 56.2% (748/1,332) of analysed sequences displayed within-patient mutation heterogeneity at more than one position in the SARS-CoV-‑2 genome, whereas 31.1% (414/1,332) had at least two such positions.

Quasi-species (≥ 2 heterogeneous positions, > 10 per variant) were most prevalent in patients who were infected with 20A/EU2 (23/47, 48.9%), 20E/EU1 (108/222, 48.6%), 20A (64/165, 38.8%), and 21K/Omicron (53/147, 36.1%), whereas remaining variants all showed a quasi-species prevalence lower than 10%, as displayed in Supplementary Table 10. On lineage-level, B.1 (34/414, 8.2%, including descendants: 287/414, 69.3%), as well as B.1.177 (20E/EU1, 93/414, 22.5%), BA.1 (21K/Omicron, 49/414, 11.8%), and B.1.1.7 (20I/Alpha, 57/414, 13.8%) with their respective sub-lineages, were most common. Among patients with > 2 heterogeneous positions, 771 mutated genomic positions were found, where 44.7% (345/771) were associated with specific SARS-CoV-2 lineages. Collectively, these findings indicate that the presence of quasi-species within patients is common, however, this information is lost when analysis is performed post-genome assembly, as only the most prevalent nt on a given genome position is reported.

All detected heterogeneous genome positions, used for the coinfection analysis, translated into 379 unique mutations, where the majority (n = 212, 55.9%) were detected in unique samples, and were mostly located in ORF1a (n = 120, 31.7%), ORF1b (n = 78, 20.6%), S (n = 79, 20.8%), N (n = 37, 9.8%), ORF3a (n = 18, 4.7%) irrespective of infecting SARS-CoV-2 variant. Fourteen mutations were in non-coding sequences, and only 0.8–3.2% were observed within ORF6, ORF10, ORF7a, ORF8, and membrane genes.

The two most prevalent quasi-species harboured amino-acid substitutions S:S943R and S:S943T, detected in 6.5% (87/1,332) and 6.3% (84/1,332) of samples, respectively, as illustrated in Supplementary Figure 7. These mutations were found in patients infected with B.1.177 (56, 48 for R and T, respectively), B.1.1.7 (32, 26), B.1 (17, 16), B.1.157 (13, 10), and other lineages. Reported data showed that S:S943T was found in B.1.351.1 sequences (14%), whereas S:S943R was found maximally in 1% of minor lineages (XCC, B.1.177.73, B.1.561) (https://outbreak.info/). Molecular docking analysis showed that S:S943T, in conjunction with S:D614G and V622F, increases binding affinity to the human ACE2 receptor [55]. The subsequent most prevalent quasi-species detected harboured the two neighbouring mutations ORF1a:A537V (n = 33, 2.5%) and ORF1a:S538A (n = 30, 2.3%), which were prevalent in 94% of AY.14 sub-lineages (https://outbreak.info/)), followed by S:A222V (n *=* 26, 2.0%) that confers improved RBD accessibility [56] (Supplementary Figure 7).

Fourteen heterogeneous positions were observed in non-coding regions, where C241T (n = 14/1,332, 1.1%) and C222T (n = 11/1,332, 0.8%) do not seem to affect the secondary structure as these are in the SL5 stem bulge. Further, C29801T (n = 13/1,332, 0.8%) and its neighbour C29802T (n = 9/1,332, 0.8%) both enlarge the large bulge at the end of the genome S2M structure.

### Risk of harbouring quasi-species according to age and immunocompromised status

We further studied whether being an older adult or being immunocompromised increased the risk of harbouring quasi-species. Immunocompromised individuals receiving solid organ transplants, cancer patients, or any patient receiving immunosuppressive or immunomodulatory therapy while, expectedly, showing > 2-fold higher odds of developing severe disease/death (OR: 2.245; 95% CI: 1.630–3.091, p < 0.001), did not have an increased likelihood of developing quasi-species (OR: 0.848; 95% CI: 0.625–1.151; p = 0.322). Similarly, individuals ≥ 60 years of age had a higher risk of severe disease/death (OR: 1.589; 95% CI: 1.117–2.259; p = 0.014) in this cohort, however, these individuals did not have a higher risk of quasi-species and instead showed an opposite trend (OR: 0.796; 95% CI: 0.631–1.003, p = 0.059). These data suggest that development of quasi-species occurs in both immunocompromised and healthy individuals.

## Discussion

In this study, we investigate in COVID-19 cohorts in four European countries, how patient and SARS-CoV-2 characteristics may contribute to severe illness. Among patients, we found that male sex, immunocompromised status, age (≥ 60 years), and ≥ 2 comorbidities were associated with higher odds of developing severe disease across all SARS-CoV-2 variants).

For SARS-CoV-2, we assessed how variants of the virus, both at clade level and through specific mutations at progressively decreasing levels of probability, impact the virus genome secondary structure, protein function, and COVID-19 severity. Mutation rates rose significantly with the emergence of VOCs, showing considerable variability across sub-genomic regions, compared with pre-Alpha variants. The increase in mutation rates might be attributed to selective pressures or to natural stochastic processes contributing to the emergence of VOCs. Because emergence of new VOCs often prompts intensified genomic surveillance that can lead to the identification of additional mutations, drawing causal inferences from these observations can be challenging. This highlights the importance of accounting for the impact of surveillance and research efforts when interpreting data on viral evolution.

Biological mechanisms underlying the association — or lack thereof — between some of the most frequent mutations and disease severity in our study, remain largely unclear. Only four hotspots with an average mutation frequency exceeding 85.0% were common across all variants. In these, a notable mutation was the non-synonymous S:D614G substitution (A23403G) that is strongly associated with VOCs that exhibit enhanced transmission and infectivity, likely contributing to the virus's rapid spread [10]. Nevertheless, the precise biological mechanism of this mutation is not fully understood. Initially, it was believed that this mutation increased binding efficacy to the ACE2 receptor on human cells. However, the prevailing view is that this mutation enhances viral infectivity by stabilising the S protein leading to higher viral loads and therefore achieving more efficient binding to the ACE2 receptor, rather than directly interfering with ACE2 binding affinity [57,58]. Notwithstanding the precise mechanisms of increased infectivity, it is also not clear why this mutation does not cause increased disease severity [57], as also observed in our study.

Conversely, mutations in structural proteins like the N protein, which were associated with up to a threefold increase in disease severity in this study, can increase viral fitness including replication and infection capabilities [26]. However, it is also possible that these mutations contribute to greater disease severity by evading the host response. We also show novel mutations in the nsp3. Nsp3 is crucial for formation and activity of the viral replication/transcription complex but also interacts with the inflammasome complex through viral and host proteins as well as RNAs [26,59]. Mutations in nsp3 linked to increased disease severity might boost viral replication and possibly allow the virus to evade host responses. In contrast, mutations in ORF3a, which plays a role in regulating apoptosis and inflammatory responses in infected cells, could contribute to more severe disease by exacerbating inflammatory responses, causing severe tissue damage and respiratory failure [60].

Moreover, most of the mutations detected in patients here, who had either mild or severe COVID-19, were linked with lineage-defining mutations among Delta and Omicron variants. It is crucial to recognise that disease severity in COVID-19 is influenced by numerous factors, including the patient's genetic makeup, overall health status, and medical history. The availability and effectiveness of treatments and vaccines also plays a significant role. Notably, during the 2021–2022 sampling period, the availability of RT-qPCR testing, patient vaccination status, and treatment policies (e.g. monoclonal antibody therapy) in the studied countries were aligned with the emergence of viral variants and evolved over time. Similarly, hospitalisation criteria changed with the emergence of new VOCs throughout the study period, impacting the population included in this study.

Finally, we show that development of quasi-species in COVID-19 patients is a common phenomenon and is not governed by their immune status. While new SARS-CoV-2 variants are suggested to emerge in immunocompromised individuals that demonstrate higher viral persistence [21], existing data also suggest that healthy individuals, including children, could also have prolonged viral persistence [20,61]. Thus, it is tempting to speculate that SARS-CoV‑2 variants in immunocompromised patients further evolve in immunocompetent individuals to tackle normal immunity for their enhanced fitness. These data add yet another layer of complexity in understanding the natural history of SARS-CoV-2 evolution and its effect on transmission and infectivity.

We recognise several limitations to our estimations of the individual impact of mutations on the overall disease phenotype. One key challenge in classifying minority variants in vivo is the sensitivity of this analysis to minority base-calls, which can lead to false-positives. In instances where multiple positions exhibit inconsistent base-calls, with each pattern aligning with different lineages, it may indicate either the presence of quasi-species of SARS-CoV-2 or co-infections with different viral strains. However, this could not be further confirmed here due to limited material available for sequencing. Additionally, some frequently observed mutations in our patients are known to enhance viral fitness, complicating the analysis of how these variants contribute to disease severity and potentially influence therapeutic outcomes (e.g. monoclonal antibody therapy) and vaccine effectiveness.

To better account for minority variants and mutations in future molecular epidemiological and viral transmission studies, we therefore recommend representing mutations as probabilities or prevalence within patient samples.

## Members of the ORCHESTRA working group

Elda Righi^3^, Alessia Savoldi^3^, Nadhem Lafhej^5^, Basil Britto Xavier^1^, Christine Lammens^1^, Davide Gibellini^3^, Michela Conti^3^, Carmine Cutone^3^, Filippo Cioli Puviani^3^, Monica Compri^3^, Romain Coppée^4^, Reyene Menidjel^4^, Maria Giulia Caponcello^8,9^, Paula Olivare Navarro^8,9^, Patricia Pérez-Palacios^8,9^, Maria Immaculada López-Hernández^8,9^

1. Laboratory of Medical Microbiology, Vaccine & Infectious Disease Institute, University of Antwerp, Antwerp, Belgium

3. Division of Infectious Diseases, Department of Diagnostics and Public Health, University of Verona, Verona, Italy

4. Université Paris Cité and Université Sorbonne Paris Nord, Inserm, IAME, Paris, France

5. AP-HP Nord, Hôpital Bichat, Department of Epidemiology Biostatistics and Clinical Research, Paris, France

8. Infectious Diseases and Microbiology Unit, University Hospital Virgen Macarena, Department of Medicine, University of Seville, Biomedicine Institute of Seville/CSIC, Seville, Spain

9. CIBERINFEC, Instituto de Salud Carlos III, Madrid, Spain

## †Current affiliation

†Infectious Diseases and Microbiology Division, Hospital Universitario Virgen Macarena Department of Medicine, University of Sevilla, Biomedicine Institute of Sevilla (IBiS)/CSIC, Sevilla, Spain.

## Supplementary Data

985000 Supplementary Information

## Supplementary Data

178000 Supplementary Tables
