# Supplementary material for: Quasi-species prevalence and clinical impact of evolving SARS-CoV-2 lineages in European COVID-19 cohorts, January 2020 to February 2022
Source: Euro Surveill. 2025 Mar 13;30(10):2400038. doi: 10.2807/1560-7917.ES.2025.30.10.2400038 (PMC11912139; doi:10.2807/1560-7917.ES.2025.30.10.2400038)
Supplement: Supplementary Information [file 2400038_SupplementaryInformation.pdf]

This supplementary material is hosted by Eurosurveillance as supporting information alongside the article **"Quasi-species prevalence and clinical impact of evolving SARS-CoV-2 lineages in European COVID-19 cohorts, January 2020—February 2022"**, *on behalf of the authors, who remain responsible for the accuracy and appropriateness of the content. The same standards for ethics, copyright, attributions and permissions as for the article apply. Supplements are not edited by Eurosurveillance and the journal is not responsible for the maintenance of any links or email addresses provided therein.*

Supplemental Results and Discussion

Secondary structure of the SARS-CoV-2 genome is influenced by nucleotide changes in intergenic regions

Nucleotide pairs wherein mutations that could have a maximum impact on secondary RNA structure were identified by screening for pairs with the highest divergence in mutation rates, i.e., where one mate had a high mutation rate whereas the other remained largely unmutated. Both mutational hotspots forming nucleotide pairs were mutated to a similar degree (99.0%). Of 8655 pairs, only 77 showed a difference in average mutation rates between the two nucleotide mates in each pair exceeding 10.0%, and in 32 pairs exceeding 20.0% (**Supplementary Figure 6C**).

Overall, pairs with highest divergence in mutation rates were located proximal to unpaired nucleotides, thus contributing to already existing opening of the RNA structure (**Supplementary Figure 6D**). Most divergent were the C3037-G3056 nucleotide pair where a ubiquitous, synonymous C3037T mutation was present at an average rate of 99%. Since the C3037-G3056 pair closes the associated RNA loop formed by these nucleotides, C3037T enlarges this loop. This substitution was consistently observed at a >85% rate across all variants in the study.

In addition to the C3037-G3056 nucleotide pair, three other asymmetrical mutation hotpots were discovered: G28,881A/T (overall mutation rate: 61%), G28,882A (35%), G28,883C (35%), translating into N:R203K/M or N:G204R predicted amino acid substitutions, respectively, and on the level of secondary structure, enlarging the neighboring bulge (starting at A28,880). The non-synonymous S:C23,604A/G mutation (average mutation rate: 60%) translates into S:P681H/R and destabilizes the 23,598-23,620 hairpin by opening a middle pair of a 3-pair helix separating two loops. Additionally, C22,995A (48%) and C10,029T (41%) substitutions are both located at the end of hairpins, thus enlarging the respective loops.

Substitution G210T (33%) located in the 5'UTR region [1-3] was predominant in the 21A/Delta (B.1.617.2) variant and is predicted to cause a loss-of-binding for spliceosome-related RNA binding serine/arginine-rich splicing factor 7 protein (SRSF7) [4]. Finally, a highly prevalent mutation observed in this study, C241T, is located in an intergenic region and likely results in an altered RNA secondary structure.

The SARS-CoV-2 genome has previously been shown to have a high propensity to form stable secondary RNA structures, similar to other highly structured RNA viruses like hepatitis C virus (HCV) [1]. Most SARS-CoV-2 mutation studies have focused on predicted amino acid substitutions, ignoring synonymous nucleotide mutations located in intergenic, non-coding regions that might influence RNA secondary structure. Although overall mutation rates in our study did not associate with nucleotide accessibility measured by nucleotide pairing within secondary structure and reactivity, we show that mutations with the largest rate divergence between the nucleotide pairs tend to lie on the edges of the existing opening in the RNA secondary structure.

Association of individual nucleotide substitutions with COVID-19 disease severity

We showed that several distinct mutations detected by the assembly and protein annotation pipeline were significantly associated with COVID-19 disease severity. Assembly followed by detection of protein level mutations constituted the highest level of probability for detected mutations, as this bioinformatics approach is the most robust, but also the least sensitive to within-sample variation.

Mutations significantly associated with severity were also abundant in N and nsp3 genes. Seven N-mutations were associated with increased disease severity, located either in the RNA-binding domain (N:V72I), the dimerization domain (N:M234I), the C-terminus (N:A376T) or affecting the linker-mediating interface with nsp3 (N:S187L, N:P199S, N:M210I, A220V) [5], where several mutations have been described previously across different variants/lineages (N:P199S in Delta/AY.70; N:S187L and N:A376T in 20A; and N:A220V in 20E/EU1). Interestingly, the nsp3 interface of the N protein also harbored mutations linked with mild disease (N:R203K, N:R203M, N:G204R, N:G215C), previously observed in 20A and 21A/Delta variants [6]. In contrast, no mutations in the interface interacting with the N protein were found in nsp3; only two substitutions (nsp3:S1206L in the beta-coronavirus-specific marker and nsp3:L1685F in the C-terminal, both novel) were associated with severe COVID-19, whereas seven mutations in the nsp3a, SARS-unique domain, marker domain, transmembrane domain, and Y domains were associated with mild COVID-19.

In addition to non-synonymous mutations identified via the protein-based SNP detection pipeline utilized in this study, an additional 46 mutations were identified using a nucleotide-based SNP detection pipeline, where 30% (14/46) showed significant association with disease severity. Three

of these mutations (ORF10:V30L [observed in 20E/EU1]; polymerase: K184N; and polymerase:775Y [insertion]) were associated with severe disease, of which the two within the polymerase have not been described previously. Among the 11 mutations associated with mild disease, S:S371F and S:S371P affected the same amino acid residue in S-RBD, three nucleotide mutations (G210T, A28,271T, G29,742T) were observed in intergenic regions, two affected ORF7a (ORF7a:V82A, ORF7a:T120I), and one substitution each was found in the envelope and nsp6 proteins (E:T9I, nsp6:G107S). S:S371F/P was observed in Omicron sub-variants and constitutes a lineage-defining mutation in BA.2 sub-lineages whereas BA.1 sub-lineages harbor S:S371L. ORF7b:T40I was found among Delta sub-lineages, and is lineage-defining for many of the lineages observed in the study (AY.122, AY.125, AY.34, AY.4, AY.4.2.3, AY.46.6, AY.98.1). Further, three mutations observed outside of coding sequences were found to have a significant negative association with disease severity, where A28,271T and G29,742T are located in the open bulge, while the G210 nucleotide forms the last closing pair of the bulge, and therefore the G210T mutation enlarges the bulge, in the SL5a steam hairpin.

Supplemental Methods

Inclusion Criteria and Ethical Approval

Overall, inclusion criteria for cases diagnosed during January 2020–February 2022 in this study included: (i) positive SARS-CoV-2 RT-qPCR diagnosis; (ii) availability of a stored nasopharyngeal swab sample from the acute phase of COVID-19 infection; (iii) availability of disease severity classified as mild, moderate, or severe COVID-19 as defined by the WHO clinical progression scale; and iv) availability of survival data within one month after diagnosis.

During January 2020–March 2021, patients with cycle threshold (Ct) values <25 in the diagnostic SARS-CoV-2 PCR were prioritized for inclusion, and if possible enrolled to fulfill 30% outpatients and 70% hospitalized patients (of which 50% died during hospitalization and 50% were not admitted to the ICU and/or had not received ventilation). From April 2021, all COVID-19 patients were enrolled in the ORCHESTRA study, irrespective of disease severity or Ct value.

Cohort-specific inclusion criteria and ethical approval in each participating country in this study is described in detail below. No study activities took place without obtaining informed consent, with the exception where a waiver was provided from the local ethical committee.

Italy

Patient enrolment was conducted through two sites: University of Verona (UNIVR) and Hospital Policlinico Sant'Orsola affiliated with the University of Bologna (UNIBO). During January 2020–March 2021, patient recruitment was conducted at UNIVR (N=349) and UNIBO (N=515) as approved by University Hospital Verona Ethics Board (3351CESC) and Comitato Etico - Area Vasta Emilia Centro (705/2021/Oss/AOUBo), respectively. Patient enrolment during April 2021–February 2022 was conducted at UNIVR (N=1387) (51COVIDCESC).

Spain

Patient enrollment was conducted through Servicio Andaluz de Salud (SAS) at Hospital Universitario Virgen Macarena (HUVM). Recruitment during January 2020–March 2021 (N=201) was conducted as approved by Comité Ético Independiente (CEI) de los Hospitales Universitarios Virgen Macarena y Virgen del Rocío (1448-N-21).

The Netherlands

Patients were recruited during January 2020–January 2022 in this study (title: Investigating SARS-CoV-2 evolution by sequencing) at the Department of Medical Microbiology and Infection Prevention, University Medical Center Groningen (UMCG, N=372). In accordance with the wavier granted by the Medical Ethics Review Board at the University Medical Center Groningen (METc-2021/505), pseudonymized patients fulfilling previously described inclusion criteria were enrolled without collection of informed consent.

France

Patient enrolment during January 2020–April 2022 was conducted at Institut National de la Sante et de la Recherche Medicale (INSERM) (N=1190) as approved by CPP Ile-de-France VI (2020-A00256-33). All collected samples were analyzed using SARS-CoV-2 RT-qPCR. Of the collected samples, 296/1190 had a Ct value <32 and were taken further for SARS-CoV-2 genome sequencing.

SARS-CoV-2 RT-qPCR

Samples from Italy (N=1403) were analyzed at the Laboratory of Medical Microbiology, University of Antwerp, Belgium (UA), where RNA was extracted from 350 µL of nasopharyngeal swab storage medium using the MagMAX Viral/Pathogen II Nucleic Acid Isolation Kit on a KingFisher Flex Purification System (ThermoFisher Scientific, The Netherlands). Subsequently, RT-qPCR (TaqPath COVID-19 CE-IVD RT-PCR, ThermoFisher Scientific) targeting three SARS-CoV-2 protein genes (S, N, and ORF1ab) was performed on a QuantStudio 5 Real-Time PCR instrument (ThermoFisher Scientific). Data analysis was performed with FastFinder Analysis software (UgenTec, Belgium), where a positive sample required detection of ≥2 target with Ct values <37, as recommended by the manufacturer.

Nasopharyngeal swab samples collected from patients in France (N=296) and Spain (N=201) and who had Ct values <32 upon COVID-19 diagnosis were analyzed at INSERM. RNA was extracted from 200 µL of sample (Total NA Isolation Kit, Roche, Switzerland) on a MagnaPure LC 2.0 System (Roche); as recommended by the manufacturer. SARS-CoV-2 RT-qPCR was performed using the RealStar® SARS-CoV-2 RT-PCR Kit 1.0 (Altona Diagnostics, Germany) targeting two genes (E gene, S gene, or non-specified). Samples were considered RT-qPCR positive if at least one gene target was detected at a Ct<37 as recommended by the manufacturer. Samples with a Ct<32 were taken forward for SARS-CoV-2 whole-genome sequencing.

Samples from the Netherlands (n= 378) were analyzed at UMCG. RT-qPCR targeting: i) the RdRp and N gene employing the AlinityM platform (Abbott Laboratories, IL, USA), and ii) the E and N gene (Gene-Xpert, Cepheid, USA) were performed directly on the nasopharyngeal swab sample as recommended by the manufacturers. RNA extraction was performed from 190 µL sample (NucliSense EasyMag, BioMérieux, France) and eluted in 110 µL as described previously [7], followed by RT-qPCR analysis targeting the E gene [8]. Samples with Ct<30 were selected and further analyzed by SARS-CoV-2 whole-genome sequencing.

SARS-CoV-2 whole-genome sequencing

Multiplexed library preparation for whole genome sequencing was performed at UA using the Illumina COVIDSeq kit (Illumina Inc., CA, USA) according to manufacturer's instructions. DNA quantification of the pooled library was performed using the Qubit dsDNA HS Assay kit (ThermoFisher Scientific). Library denaturation was performed using the NextSeq 550/500 High

Output kit v2 with a 1.4 nM Phix Library positive control with 1% spike-in. Paired-end sequencing (2x74 bp) was performed on a NextSeq 500/550 instrument (Illumina Inc.).

At INSERM, library preparation was performed utilized the NEBNext ARTIC SARS-CoV-2 Companion Kit (Oxford Nanopore Technologies, New England Biolabs, MA, USA), following the ARTIC protocol. Briefly, nucleic acid extraction (MagNA Pure LC Total Nucleic Acid Isolation Kit, Roche) and reverse transcription was performed with LunaScript and random hexamers. Target amplification was performed with two primer pools (ARTIC nCoV-2019 V3 panel), followed by library preparation (NEBNext Companion Module, Ligation Sequencing, SQK-LSK 109) and sequenced with MinION R9.4.1 flow cells on a GridION instrument (Oxford Nanopore Technologies, United Kingdom).

At UMCG, the EasySeq™ RC-PCR SARS-CoV-2 WGS kit (NimaGen BV) was used. RNA was extracted from 190 µL of sample (NucliSense EasyMag, bioMérieux, France), followed by cDNA conversion (LunaScript RT supermix (5x), New England Biolabs). 10 µL cDNA was split into two reactions with separate primer / probe pools used for targeted amplification with the RC-probe 32. Amplicons were then pooled based on the initial Ct-value according to the manufacturer. Next, 9 pM final Library pools were sequenced on an Illumina MiSeq platform using a Mid Output Kit (2x200 cycles PE, Illumina Inc., CA, USA).

SARS-CoV-2 variant classification

Raw Illumina NextSeq data quality assessment was performed using FastQC, followed by quality trimming with TrimGalore v. 0.6.7 (https://github.com/FelixKrueger/TrimGalore), reference mapping against the SARS-CoV-2 genome (GenBank: NC_045512.2) using the CLC Genomics Workbench v.9.5.3 (Qiagen), and extraction of consensus sequences. MiSeq data were processed with MiSeq control software v2.4.0.4 and MiSeq Reporter v2.4. Generated Fastq files were imported into CLC Genomics Workbench v21.0.4 (Qiagen, Germany) where raw reads were trimmed and mapped against SARS-CoV-2 reference (GenBank: MN908947.3). Nanopore sequencing data from INSERM were managed using a specific workflow using the Medaka-based Artic-nCoV workflow v1.1.0 adapted by EPI2ME lab (rev. 41f235cdf1). Clade and lineage assignment was performed for all SARS-CoV-2 consensus sequences using Nextclade v.2.2.0 (https://clades.nextstrain.org) and Pangolin v1.9 (4.0.6) (https://pangolin.cog-uk.io), respectively.

Supplemental Table Legends

Supplementary Table 1. Structure of the SARS-CoV-2 genome as described by Finkel *et al.* [9] using the Wuhan-Hu-1 genome (GenBank: NC_045512.2) as reference. A frameshift within the polymerase gene of ORF1ab results in a split into ORF1a and ORF1b for some analyses within the study.

Supplementary Table 2. Overview of the most dominant Pangolin SARS-CoV-2 lineages within different NextStrain clades in the study. Only lineages with a total abundance exceeding 1.0% are displayed.

Supplementary Table 3. Characteristics of enrolled COVID-19 patients in the study by infecting SARS-CoV-2 variant. Disease severity was classified according to the WHO disease severity scale [10]. IQR: inter-quartile range. ns: non-significant. na: data not recorded. ICU: intensive care unit. Ct: cyclic threshold.

Supplementary Table 4. Association of patient characteristics, SARS-CoV-2 viral variants, and COVID-19 disease severity (N=1762). OR: odds ratio. aOR: adjusted OR. CI: confidence interval. ns: non-significant. [a] bivariate mixed model with random intercept by country. [b] multivariate mixed model with random intercept by country (variance=0.8553, 4 countries), N=1313. [c] ≥2 comorbidities (cardiovascular disease, diabetes, chronic pulmonary disease, chronic kidney disease, chronic liver disease, psychiatric disease, neurologic disease, autoinflammatory disease, immunosuppression, HIV, transplant, cancer).

Supplementary Table 5. Association of patient characteristics, SARS-CoV-2 viral variants, and hospitalization (N=1661). OR: odds ratio. aOR: adjusted OR. CI: confidence interval. ns: non-significant. NE: not possible to estimate. [a] bivariate mixed model with random intercept by cohort. [b] multivariate mixed model with random intercept by cohort (variance=0.9806, 4 cohorts), N=1,267. [c] ≥2 comorbidities (cardiovascular disease, diabetes, chronic pulmonary disease, chronic kidney disease, chronic liver disease, psychiatric disease, neurologic disease, autoinflammatory disease, immunosuppression, HIV, transplant, cancer). Note that the French cohort did not have any mild cases and was therefore excluded from this analysis.

Supplementary Table 6. Pairwise comparisons of viral loads expressed as combined cyclic threshold (Ct) values of patients infected with different SARS-CoV-2 variants in the study

(N=1531). Statistical comparisons were performed using pairwise Wilcoxon tests followed by Bonferroni post-hoc correction. Ns: non-significant. CI: confidence interval.

Supplementary Table 7. Mutation hotspots in the SARS-CoV-2 genome identified in this study. Genome positions refer to the Wuhan-Hu-1 genome sequence (GenBank: NC_045512.2).

Supplementary Table 8. Association of individual nucleotide or amino acid substitutions in the SARS-CoV-2 genome with disease outcome (N=1332). OR: odds ratio. aOR: adjusted OR. CI: confidence interval. ns: non-significant. NE: not possible to estimate. [a] FDR-correction for multiple testing by coding region (S, M, N, ORF1ab, other).

Supplementary Table 9. Average mutation rates of observed nucleotide or amino acid substitutions in infecting SARS-CoV-2 variants (N=1332) within the study. Mutations are described using the Wuhan-Hu-1 genome (GenBank: NC_045512.2) as reference.

Supplementary Table 10. Number of samples with at least one or two heterogeneous positions in the SARS-CoV-2 genome. Distribution of samples with exactly one or two heterogeneous positions in the SARS-CoV-2 genome separated by dominant / infecting SARS-CoV-2 variant.

Supplemental Figures

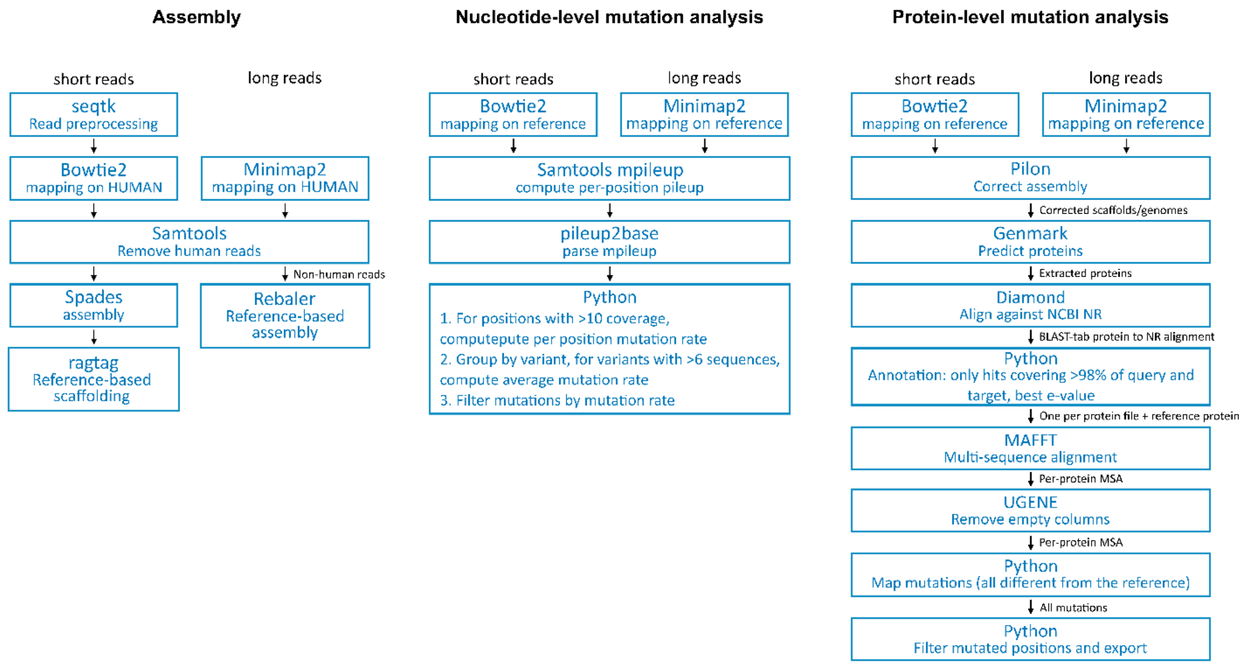

Supplementary Figure 1. Schematic overview of bioinformatic analyses performed for detection of mutations in the SARS-CoV-2 genome.

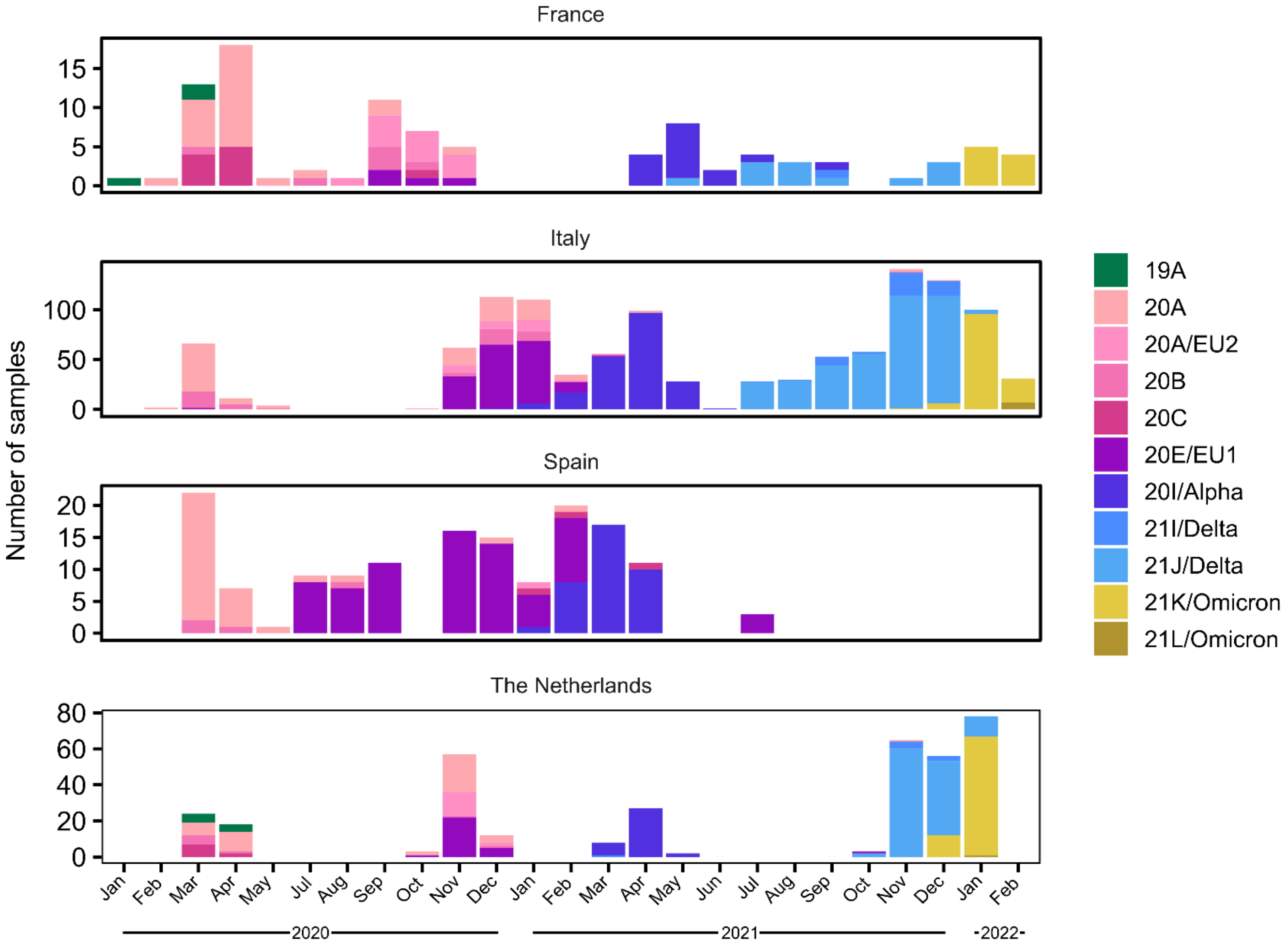

Supplementary Figure 2. Distribution of SARS-CoV-2 variants over time in enrolled patients with COVID-19 across the different participating countries (*N*=1762).

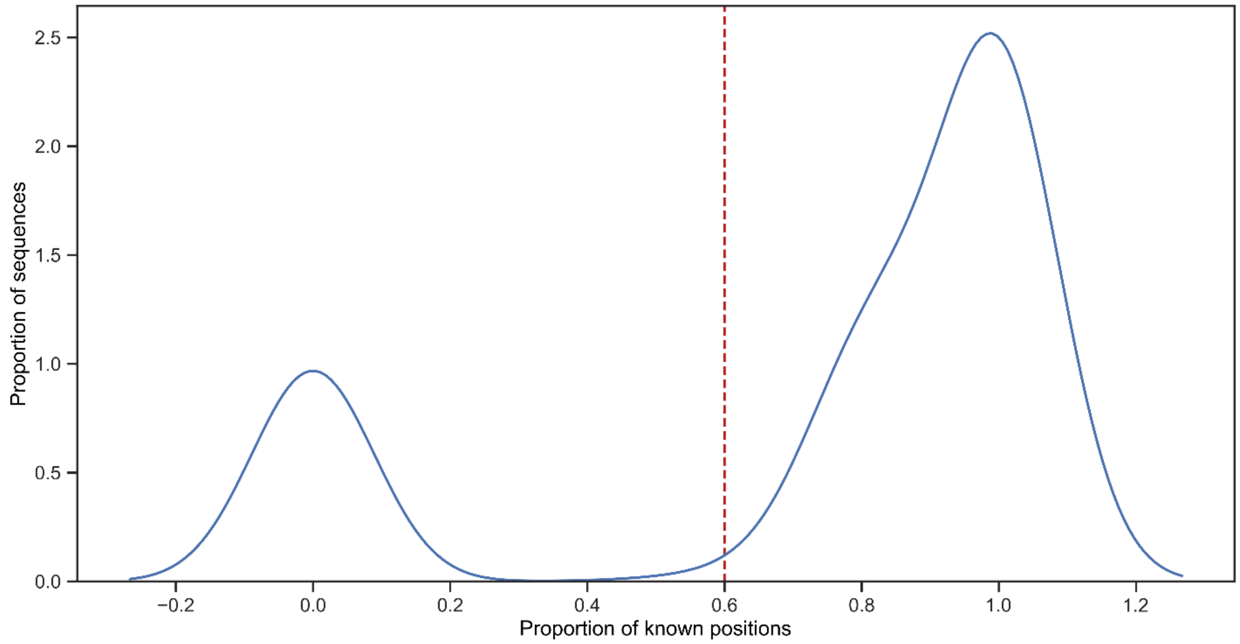

Supplementary Figure 3. Distribution of proportion of known mutation positions across samples. Samples with low proportion of known mutations (left of the red line) were removed from downstream analysis (*N*=430) and resulted in a final analysis population of *N*=1332.

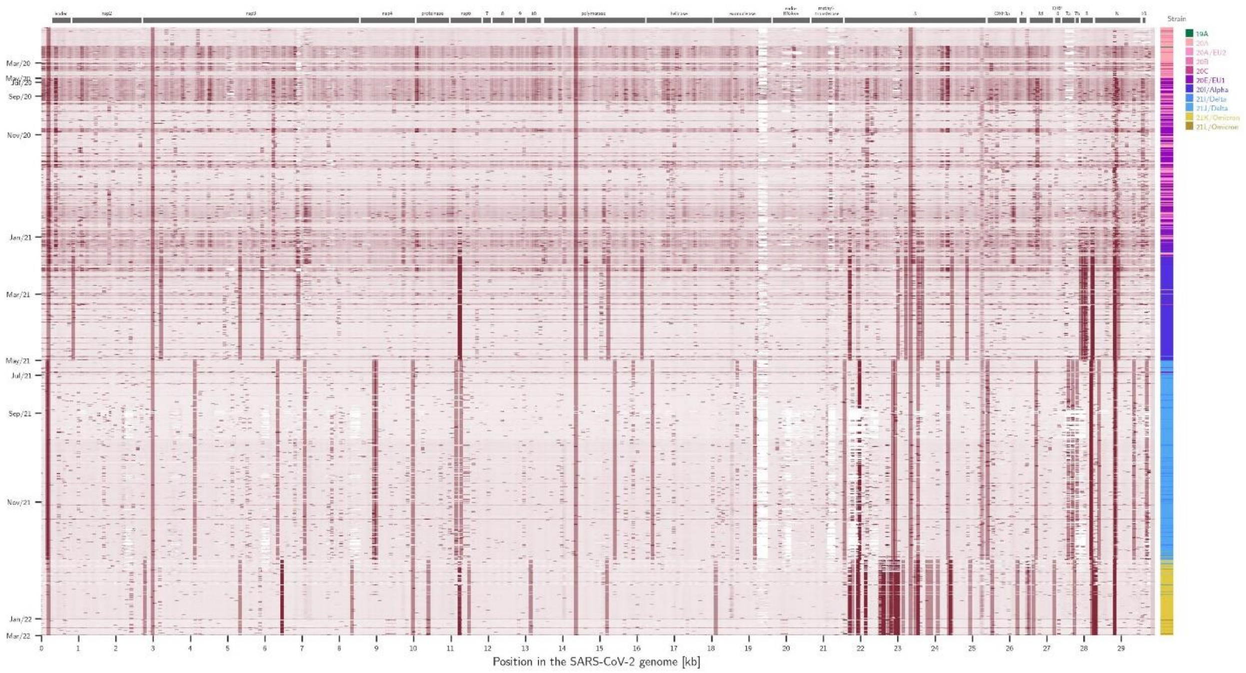

Supplementary Figure 4. Heatmap presenting the per-position mutation rate across the SARS-CoV-2 genome. The x-axis corresponds to the nucleotide position in the SARS-CoV-2 genome, and the y-axis to individual sequences, ordered by date. Coding regions of the SARS-CoV-2 genome are indicated above the heatmap. Mutation rates were averaged over a 100 bp window, where at least 20 values within each window were known for the plot. The panel on the right indicates the SARS-CoV-2 variant classification for each sample.

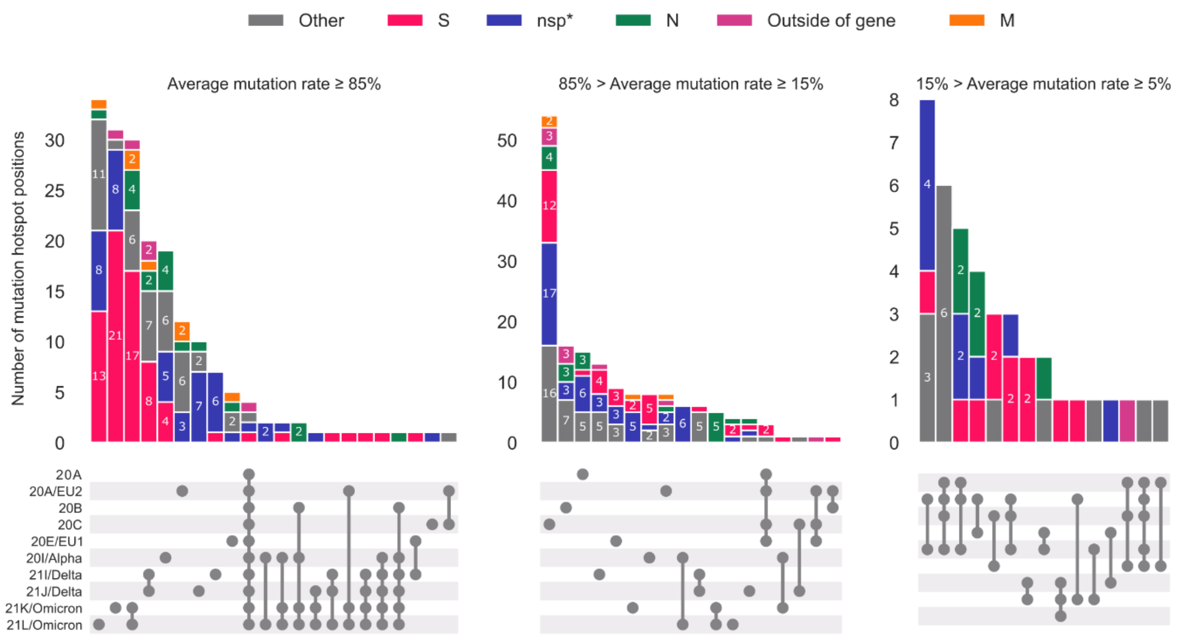

Supplementary Figure 5. Number of mutation hotspots across three levels of mutation rate, divided by the position on the genome. Bottom panels correspond to the lineages the mutations were detected. Connections show the mutations detected in multiple variants. Top panels show the stack charts of the mutations divided by their location. nsp*: nsp2. 3, 4, 6, 9, and 10.

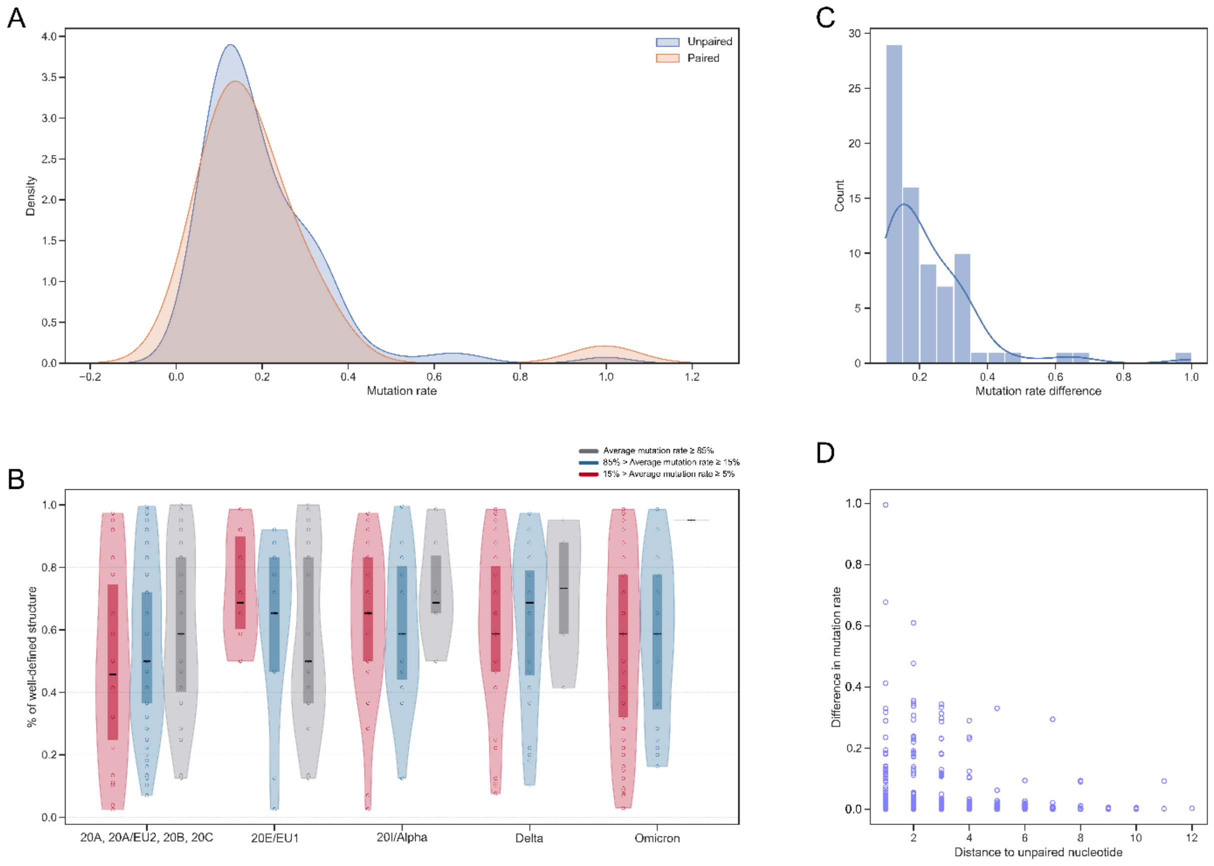

Supplementary Figure 6. Average mutation rate in respect to the genome secondary structure. (A) Kernel density plot displaying the proportion of mutations across average mutation rates for paired and un-paired nucleotides. **(B)** Violin plot displaying the distribution of proportion of well-defined structures for the mutation hotspots as defined in **Figure 4**. Box plots show median (line), 25th–75th percentiles (box), and outliers (circles). Violin plots implement a rotated kernel density plot on each side, adding information regarding distribution of the measured data; the width of the violin indicates the frequency. **(C)** Histogram of the absolute differences in the average mutation rate between nucleotides creating a pair in the RNA secondary structure. **(D)** Absolute differences in the mutation rate with respect to the distance from the nearest unpaired nucleotide.

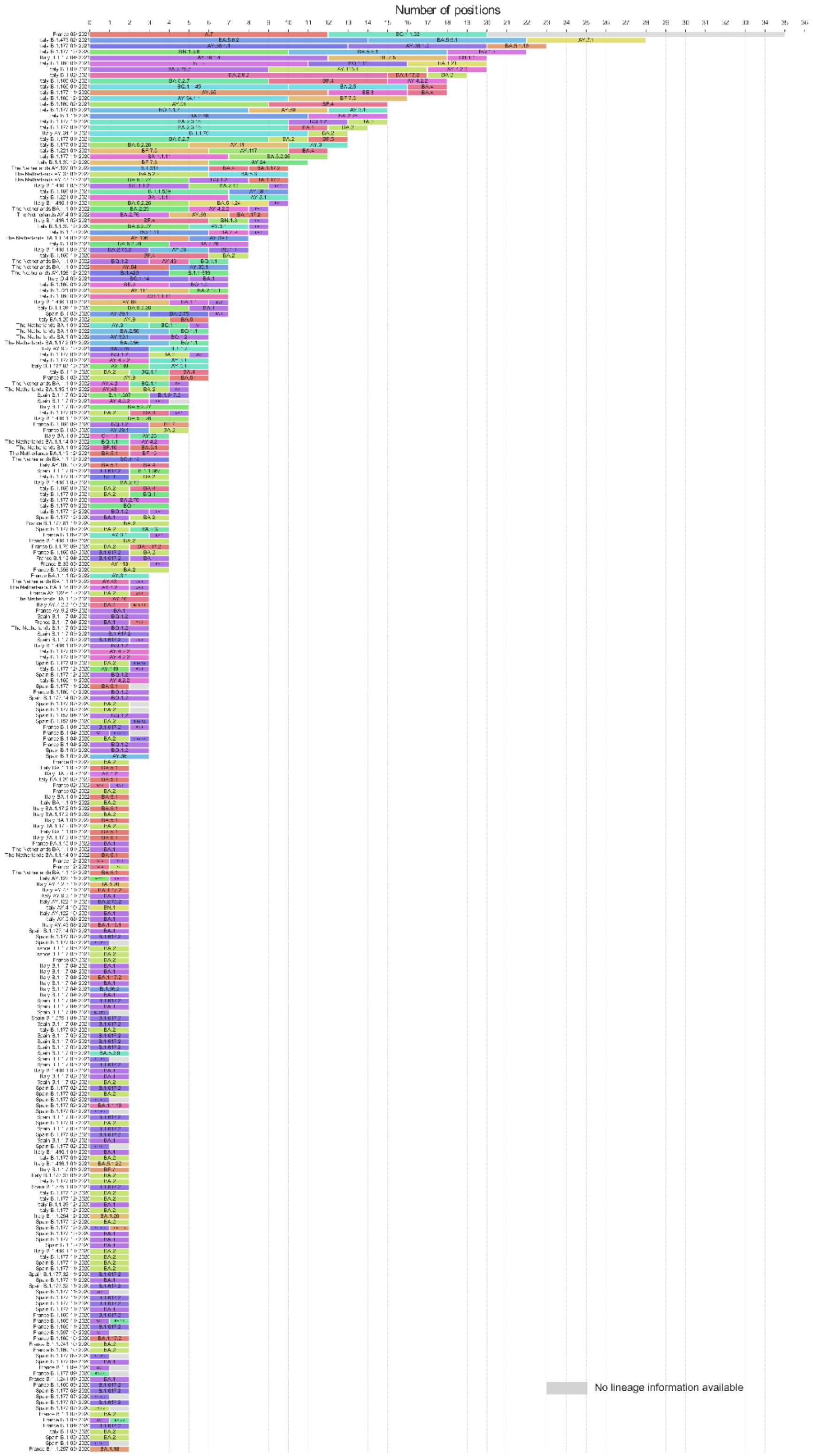

Supplementary Figure 7. Bar plot indicating number of heterogeneous positions per sample and their defining Pangolin lineage. Country of origin, dominant infecting SARS-CoV-2 variant, and collection month/year are displayed on the y-axis.

References

1. Tavares, R.C.A., et al., *The global and local distribution of RNA structure throughout the SARS-CoV-2 genome.* J Virol, 2021. 95(5).
2. Cao, C., et al., *The architecture of the SARS-CoV-2 RNA genome inside virion.* Nat Commun, 2021. 12(1): p. 3917.
3. Wu, F., et al., *A new coronavirus associated with human respiratory disease in China.* Nature, 2020. 579(7798): p. 265-269.
4. Horlacher, M., et al., *A computational map of the human-SARS-CoV-2 protein-RNA interactome predicted at single-nucleotide resolution.* NAR Genom Bioinform, 2023. 5(1): p. lqad010.
5. Khan, M.T., et al., *SARS-CoV-2 nucleocapsid and Nsp3 binding: an in silico study.* Arch Microbiol, 2021. 203(1): p. 59-66.
6. Cong, Y., et al., *Nucleocapsid Protein Recruitment to Replication-Transcription Complexes Plays a Crucial Role in Coronaviral Life Cycle.* J Virol, 2020. 94(4).
7. Cassidy, H., et al., *Evaluation of the QIAstat-Dx RP2.0 and the BioFire FilmArray RP2.1 for the Rapid Detection of Respiratory Pathogens Including SARS-CoV-2.* Front Microbiol, 2022. 13: p. 854209.
8. Corman, V.M., et al., *Detection of 2019 novel coronavirus (2019-nCoV) by real-time RT-PCR.* Euro Surveill, 2020. 25(3).
9. Finkel, Y., et al., *The coding capacity of SARS-CoV-2.* Nature, 2021. 589(7840): p. 125-130.
10. WHO Working Group on the Clinical Characterisation Management of, C.-i., *A minimal common outcome measure set for COVID-19 clinical research.* Lancet Infect Dis, 2020. 20(8): p. e192-e197.
